# Caribbean *Bulimulus* revisited: physical moves and molecular traces (Mollusca, Gastropoda, Bulimulidae)

**DOI:** 10.7717/peerj.1836

**Published:** 2016-03-29

**Authors:** Abraham S.H. Breure

**Affiliations:** Naturalis Biodiversity Center, Leiden, Netherlands; Royal Belgian Institute of Natural Sciences, Brussels, Belgium

**Keywords:** Orthalicoidea, Distribution, Central America, Ecuador, West Indies, Florida, Alien species, Phylogenetics

## Abstract

Twenty-five samples of *Bulimulus* species are studied, partly from localities within their known distribution range, partly based on interceptions where the material originates from localities where the species seem to be recently introduced and non-native. Molecular study of cytochrome oxidase 1 (CO1) reveals the origin of some of these introductions, but is less conclusive for others. Four different methods for species delimitation were applied, which did not result in unambiguous species hypotheses. For a rapid identification of morphologically indistinct species, a more comprehensive database of sequences is needed.

## Introduction

The genus *Bulimulus* is widespread in the Caribbean region, and on the mainland of Central America; also a number of species do occur in dispersed regions of South America (Breure, 1974; Breure & Borrero, 2008; Linares & Vera, 2012; Cuezzo, Miranda & Constanza Ovando, 2013; Ramírez, Paredes & Arenas, 2003; Simone, 2006; Thompson, 2011). Breure (1974) presented a revision of the Caribbean species, with a key to species, based on external shell morphology only. One of the most common species, *Bulimulus guadalupensis* (Bruguière, 1789), was reported then mainly from the Windward Group and supposedly imported on Jamaica, and Hispaniola. Shortly afterwards it was reported as being introduced on Curaçao (Breure, 1975). This very variable species, however, did not show infraspecific differentiation using measurements of the shells (Breure, 1974: 21). Another Caribbean species, *B. diaphanus* (Pfeiffer, 1855), was divided into two subspecies ranging from Hispaniola to St. Croix, and from St. Martin to Iles des Saintes, respectively (Breure, 1974). Anatomical data of *Bulimulus* species are scarce (Breure, 1978; Miquel, 1991) and mainly concern the mainland species. Molecular data has become available during the past decades, the most extensive treatment of this genus by Breure & Romero (2012) provides seven sequences, of which five are from the Caribbean. In order to obtain more insight in phylogenetic relations and zoogeographical patterns, additional molecular data from *Bulimulus* species throughout the distribution range are needed. In recent years reports of introduced *Bulimulus* taxa throughout the Caribbean and in the USA have increased (D Robinson, pers. comm., 2014). The author frequently received photographs of *Bulimulus* species for identification. Species of this genus—for which more than 100 names are available (Breure, 1979)—typically possess few reliable external characters, and are thus difficult to recognise from a photograph alone. Most of the photographs were from material captured by the US Dept of Agriculture (USDA) at border sites, which were considered alien species, often with unclear provenance. Moreover, some Florida populations of *Bulimulus* have not been well documented as yet.

The aim of this paper is provide additional molecular and occurrence data on Caribbean (sensu lato, incl. Florida) *B. guadalupensis* and *B. diaphanus*, and to test whether recent imports of *Bulimulus* species in the USA can be traced back to a South American origin. A second aim is to test whether different species delimitation methods may be helpful to untangle morphologically very similar species (cf. Prévot et al., 2013).

## Material and Methods

Intercepted or collected material of *Bulimulus* species was received originating from Argentina, Bahamas, Barbados, Colombia, Dominica, Dominican Republic, Ecuador, Guadeloupe, Haiti, Honduras, Jamaica, Paraguay, St. Kitts and Nevis, and the USA Collection data, accession numbers for museum vouchers and GenBank accession numbers of all sequenced material are listed in Table 1. All tissue samples were taken from snail feet and transferred to 96% ethanol. Whole genomic DNA was extracted with DNeasy kit (Quiagen Inc.) following the manufacturer’s protocol. Fragments of mitochondrial cytochrome oxidase 1 (CO1) were amplified using the Folmer primers (Folmer et al., 1994), as described in Breure & Romero (2012). New sequences obtained during the present study are indicated in Table 1. Sequences were aligned using MAFFT as implemented in Geneious 7.1.3 (Biomatters Ltd.). The substitution model selected by jModeltest 2.1 (Posada, 2008), using the Akaike Information Criterion, was GTR+ I+ G.

**Table 1:** Material used in this paper.

MOTU	LabID	Species			Voucher	Locality				Collector(s)	CO1
s1	FL_1301	Bulimulus	sporadicus	(d’Orbigny, 1835)	RMNH.MOL. 336660	USA	Florida	Nassau County	S Callahan	H.Lee& EW Cavin	KP117235
	AR_1308	Bulimulus	gracilis	(Hylton Scott, 1948)	IML-BD368	Argentina	Salta		Sierra Colorada, Pala Pala	E. Salas O.	KP117237
s2	PA_1316	Bulimulus	sporadicus	(d’Orbigny, 1835)	RMNH.MOL. 336661	Paraguay	Concepcion		Garay-Cue	U. Drechsel	KP117238
	CO_1414	Bulimulus	sp.		CMC-FJB78	Colombia	Magdalena		Santa Marta, Corr. Gayra, Vereda Mosquito	FJ Borrero	KP117236
gA	PR_1707	Bulimulus	guadalupensis	(Bruguiere 1789)	USDA 110196	Puerto Rico		Huamaco	Isla Vieques, Barrio Punta Arenas, Monte Pirata	DGRobinson	KP117239
gA	PR_1708	Bulimulus	guadalupensis	(Bruguiere 1789)	USDA 110199	Puerto Rico		Huamaco	Isla Vieques, Barrio Florida	DGRobinson	KP117249
gA	PR_1709	Bulimulus	guadalupensis	(Bruguiere 1789)	USDA 110195	Puerto Rico		Bayamon	Guayabo, Amelia Industrial Park	DGRobinson	KP117246
gA		Bulimulus	guadalupensis	(Bruguiere 1789)	RMNH. MOL. 106983	Dominican Republic			Santo Domingo	J. Grego	JF514630
gB	DR_1710	Bulimulus	guadalupensis	(Bruguiere 1789)	USDA 110201	Dominican Republic		Samana	Las Terrenas	DGRobinson	KP117253
gA	DR_1711	Bulimulus	guadalupensis	(Bruguiere 1789)	USDA 110197	Dominican Republic		Samana	Come Pan	DGRobinson	KP117244
gA	DR_1712	Bulimulus	guadalupensis	(Bruguiere 1789)	USDA 110198	Dominican Republic		Espaillat	La Cantera	DGRobinson	KP117241
gA	DR_1714	Bulimulus	guadalupensis	(Bruguiere 1789)	USDA 110200	Dominican Republic			4 km S Cabrera, on road Nagua-Cabrera	DGRobinson	KP117245
gA	FL_1717	Bulimulus	guadalupensis	(Bruguiere 1789)	USDA 110208	USA	Florida	Dade	Miami, Coral Gables	DGRobinson	KP117240
gB	BA_1720	Bulimulus	guadalupensis	(Bruguiere 1789)	USDA 110207	Barbados	St.Thomas		Sandy Lane	DGRobinson	KP117250
gA	DO_1722	Bulimulus	guadalupensis	(Bruguiere 1789)	USDA 100706	Dominica	St.George		Bellevue Chopin	DGRobinson	KP117242
gA	DO_1724	Bulimulus	guadalupensis	(Bruguiere 1789)	USDA 100713	Dominica	St. Luke		Pointe Michel, Morne Lofty	DGRobinson	KP117243
gB	GU_1725	Bulimulus	guadalupensis	(Bruguiere 1789)	USDA 110209	Guadeloupe	Grande-Terre		Sainte-Anne	DGRobinson	KP117252
gB	JA_1727	Bulimulus	guadalupensis	(Bruguiere 1789)	USDA 110194	Jamaica		Hanover	Lucea, western Lucea Harbor	DGRobinson	KP117251
gA	EC_1728	Bulimulus	guadalupensis	(Bruguiere 1789)	USDA 110210	Ecuador			(USDA interception)	A Hansen	KP117247
gA	HO_1729	Bulimulus	guadalupensis	(Bruguiere 1789)	USDA 110162	Honduras		ElParaiso	Yuscaran, km 50 road Tegucigalpa-Danli	H Deschamps	KP117248
**d1**		Bulimulus	diaphanus	(Pfeiffer, 1855)	RMNH.MOL.114173	Jamaica		St. Ann	Runaway Bay	DGRobinson	KP117232
**d2**		Bulimulus	diaphanus	(Pfeiffer, 1855)	RMNH.MOL.114174	St. Kitts & Nevis	Nevis		Herbert Heights	DGRobinson	KP117233
**d1**		Bulimulus	diaphanus	(Pfeiffer, 1855)	RMNH.MOL.114274	Haiti			(USDA interception)	D. Valleso	KP117231
**d3**		Bulimulus	diaphanus	(Pfeiffer, 1855)	ANSP A22054	Bahamas			(USDA interception)	G Watkins	JF514633

**Notes.**

Abbreviations for vouchers ANSP Academy of Natural Sciences, Philadelphia, USA CMC Cincinnati Museum, Cincinnati, USA IML Instituto Miguel Lillo, Tucumán, Argentina RMNH Naturalis Biodiversity Center (NBC), Leiden, the Netherlands UF Florida Museum of Natural History, Gainesville, USA USDA United States Department of Agriculture, Malacology Collection, ANSP, Philadelphia, USA

Other abbreviations LabID NBC molecular laboratorium code, including a two-letter locality code MOTU molecular operational taxonomic unit sensu (Blaxter, 2004)

Phylogenetic trees were inferred by application of Neighbor-Joining (NJ), Maximum Likelihood (ML), Maximum Parsimony (MP), and Bayesian Inference (BI) methods. NJ trees were constructed using MEGA6 (Tamura et al., 2013) with Kimura 2-parameter (K2P) and 500 bootstrap replicates. ML trees were inferred using PhyML 3.0 (Guindon et al., 2010), with four substitution rate categories considered, and gamma shape parameters, transition/transversation ratios, and nucleotide frequencies were estimated from the data. Nodal support of topologies was inferred by calculating aLRT statistics. MP trees were constructed using MEGA6 (Tamura et al., 2013), resulting in a most parsimous tree with length = 906; the consistency index is 0.445100 for all parsimony-informative sites. The tree was obtained using Subtree-Pruning-Regrafting and 500 bootstraps. BI trees were constructed using MrBayes 3.2.2 (Ronquist & Huelsenbeck, 2003), based on a cold chain and three incrementally heated chains (*T* = 0.2), running for 1,100,000 generations with a sample frequency of 200. The burn-in was 25% and the remaining trees were used for building a consensus tree and calculating the Bayesian posterior clade probabilities (Larget & Simon, 1999). Both ML and BI software were used as implemented in Geneious 7.1.3 (Biomatters Ltd.). All trees were rooted with an outgroup of *Drymaeus vexillum* (Broderip, 1832) and *Neopetraeus tessellatus* (Shuttleworth, 1854), for which sequences were retrieved from GenBank (Breure & Romero, 2012). Branch support was considered as well-supported if higher than 70 (bootstrapping: bs), resp. 0.9 (posterior probabilities: pp).

Evolutionary analysis of the original alignment was further explored by using SplitsTree4 v. 4.12.6 (Huson & Bryant, 2006). This program produces phylogenetic graphs with split networks; the K2P parameter was used in combination with the Neighbor-Net setting. Species delimitation was investigated using different methods, both with the original alignment and with a subset using only *B. guadalupensis*, *B. diaphanus*, and *B. sporadicus*: (1) Classical barcode gap analysis (BGA) using K2P-distances (a) 3% threshold, (b) 4% stylommatophoran threshold (both methods following Prévot et al., 2013, who also cite references debating the use of K2P); (2) Species Delimiting as implemented in Geneious 7.1.3. (Biomatters Ltd.) (SDG); (3) Genealogical Sorting Index (GSI); (4) Automated Bar code Gap Discovery (ABGD).

BGA was explored using Estimates of Evolutionary Divergence between Sequences (EDS) and estimates of Net Evolutionary Divergence between Group of Sequences (NEDGS). These were conducted in MEGA6 (Tamura et al., 2013) by calculating the number of base substitutions per site from between sequences. Standard error estimate(s) were obtained by a bootstrap procedure (500 replicates). Analyses were conducted using the Maximum Composite Likelihood model (Tamura, Nei & Kumar, 2004). Codon positions included were 1st+2nd+3rd+ Noncoding and all positions containing gaps and missing data were eliminated, resulting in 585 positions in the final dataset analysed.

The SDG plug-in in Geneious v. 7.1.3 allows evaluating the phylogenetic exclusivity of each putative species interpreted as a clade by testing the probability that this exclusivity or monophyly has occurred by chance in a coalescent process. It further assesses the probability with which a putative species can be diagnosed successfully on a phylogenetic tree by comparing intra- and interspecific genetic distances and its ration. SDG calculates values of P ID(strict), i.e., the mean probability and its confidence interval of making a correct identification of an unknown specimen of a clade (not as sister of this clade) using placement on a tree (Masters, Fan & Ross, 2011). The method also calculates Rosenberg’s P_AB_ (Rosenberg, 2007), a test for taxonomic distinctiveness based on the null hypothesis that monophyly is a chance outcome of random branching, and Rodrigo’s P (Randomly Distinct) (Rodrigo et al., 2007), which is the probability that a clade has the observed degree of distinctiveness due to random coalescence.

The GSI method quantifies the historical relationships among groups of (putative) taxa by measuring the exclusive ancestry of a group using a rooted tree topology. A group is defined as a set of commonly labeled branch tips and exclusivity is the amount of ancestry for a group that is common to only members of the group, measured on a scale (the index), with a level of support (*p*-value). In the initial stages of divergence the values are at or near 0, at the final stages of genealogical sorting the values reach 1, representing exclusive ancestry (i.e., monophyly) (Cummings, Neel & Shaw, 2008).

The ABGD method sorts the sequences into hypothetical species based on the barcode gap, which can be observed whenever the divergence among organisms belonging to the same species is smaller than divergence among organisms from different species. ABGD uses a range of prior intraspecific divergence to infer from the data a model-based one-sided confidence limit for intraspecific divergence. The method then detects the barcode gap as the first significant gap beyond this limit and uses it to partition the data. The aligned dataset was uploaded to the website (http://wwwabi.snv.jussieu.fr/public/abgd/abgdweb.html) and run with K2P distances under default settings. These settings were a Prior Intraspecific divergence ranging from Pmin = 0.001 to Pmax 0.1 in ten steps, a relative gap width *X* = 1.5, and a distance distribution Nb bins = 20 (Puillandre et al., 2012).

## Results

### Occurrence data

For the most common Caribbean *Bulimulus* species, *B. guadalupensis* (Bruguière, 1789), new introductions were newly traced back to the following Neotropical countries: Ecuador (USDA 110210), Costa Rica (USDA 110245), and Honduras (USDA 110162). All introductions were signaled via an ‘U-loop’, viz. the interception at USA-borders testified that the species was present in the country of departure of the flight or shipment concerned (in all cases no stop-overs were made). However, precise locations within those countries were difficult to pinpoint. Another new record for a population of this species was in Florida, Dade County, Miami, near Coconut Grove (USDA 110205-6), and near Coral Gables (USDA 110208), where specimens have been collected by D.G. Robinson, in respectively 1999 and 2010. The status of *B. guadalupensis* on Jamaica was confirmed as “introduced by humans” by (Rosenberg & Muratov (2006): 140); see also under Phylogenetic analyses.

Another introduction of *Bulimulus* in Florida concerns a species that was originally reported from Jacksonville by B. Frank and H. Lee in 2009 (H Lee, pers. comm., 2010). Because the original spot was near a container company, it was surmised to be an alien species from an unknown source area. Additional surveys in Jacksonville soon revealed that this then unidentified species was present in other spots as well and that its dispersal has taken place by rail. So far more than 20 populations have been discovered in and around Jacksonville along railroads, and far as 300 km as the crow flies SSW of Jacksonville. The species was identified by ANSP-USDA as *B. sporadicus* (d’Orbigny, 1835) (Frank & Lee, 2014), matching with specimens identified earlier as such from Houston, Texas (RMNH 114266) (D Robinson, pers. comm., 2014; Breure & Romero, 2012). This species is referred to in the present paper as *B*. cf. *sporadicus*, see ‘Discussion’.

### Phylogenetic analyses

**Figure 1:**
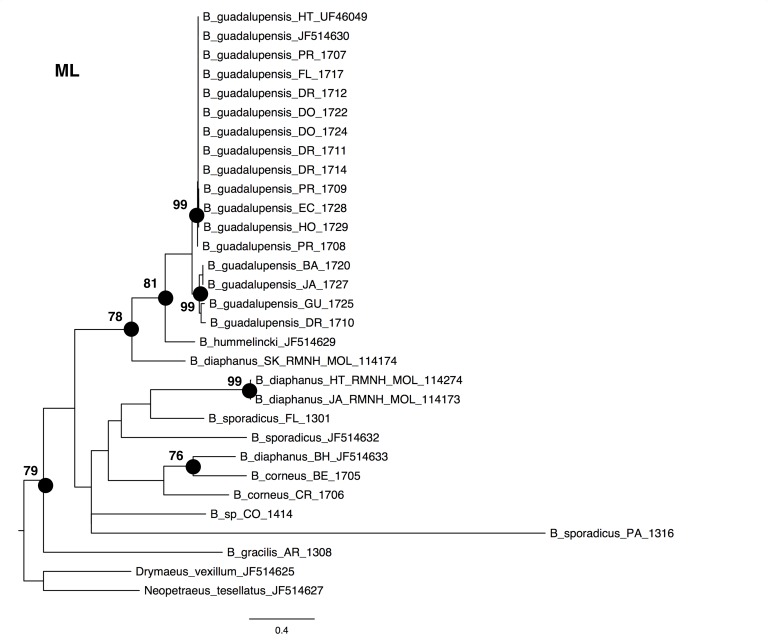
Maximum-likelyhood phylogeny for *Bulimulus* species, based on 654 bp cytochrome oxidase I mitochondrial DNA. Bootstrap values of 70 and above are presented to the left of the nodes indicated by black dots. Scale bar in substitutions/site.

**Figure 2:**
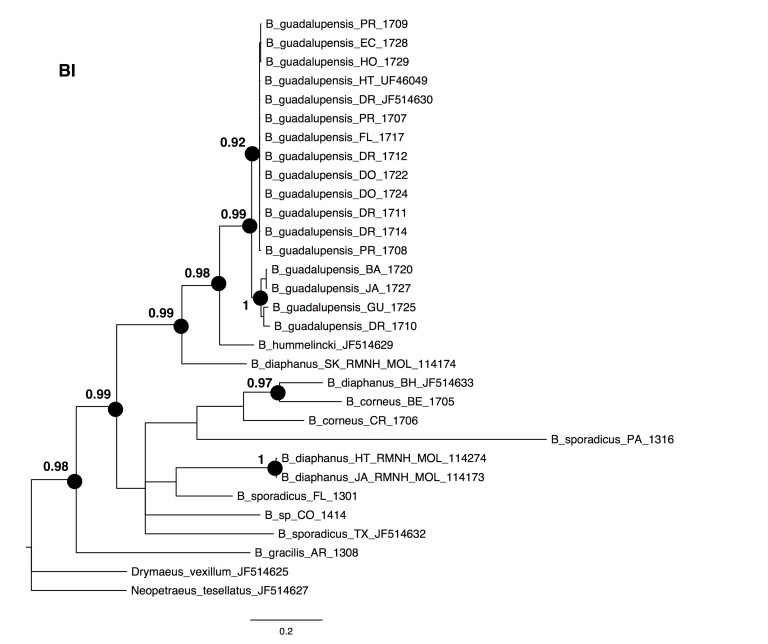
Bayesian phylogeny for *Bulimulus* species using MrBayes, based on the same dataset as shown in Fig. 1. Posterior probabilities of 0.9 or above are indicated by black dots at the nodes. Scale bar in substitutions/site.

The NJ, ML and BI trees resulted in similar topologies (Figs. 1 and 2; NJ: Supplementary information Fig. 1). The 17 sequences of *B. guadalupensis* fell into two sister groups that were both well-supported (bs: 100 (NJ), (ML); pp: 1.0 and 0.92 respectively). The MP tree (Fig. S2) also supports the division of *B. guadalupensis* into two groups (bs: 99), and suggests that the Florida population is likely from a Puerto Rico or Hispaniola source population. In the MP tree the clade with sequences from Puerto Rico (Guayabo), Honduras and Ecuador had relatively strong support (bs: 87). *B. hummelincki* and *B. diaphanus* from St. Kitts appeared as paraphyletic in the same clade (bs: 71 (NJ), 78 (ML); pp: 0.99), which was sister group to all other *Bulimulus* species analysed in this study, except *B. gracilis* which was basally in all topologies. Within this second, weakly supported clade different topologies were seen, with polytomies in ML and BI. However, all methods suggest that *B. diaphanus* intercepted from the Bahamas is more closely related to the Central American *B. corneus* than to the other sequences of *B. diaphanus.* The latter are not a homogenous group, however, as the Nevis specimen was slightly different from the Jamaican/Haitian specimens.

**Figure 3:**
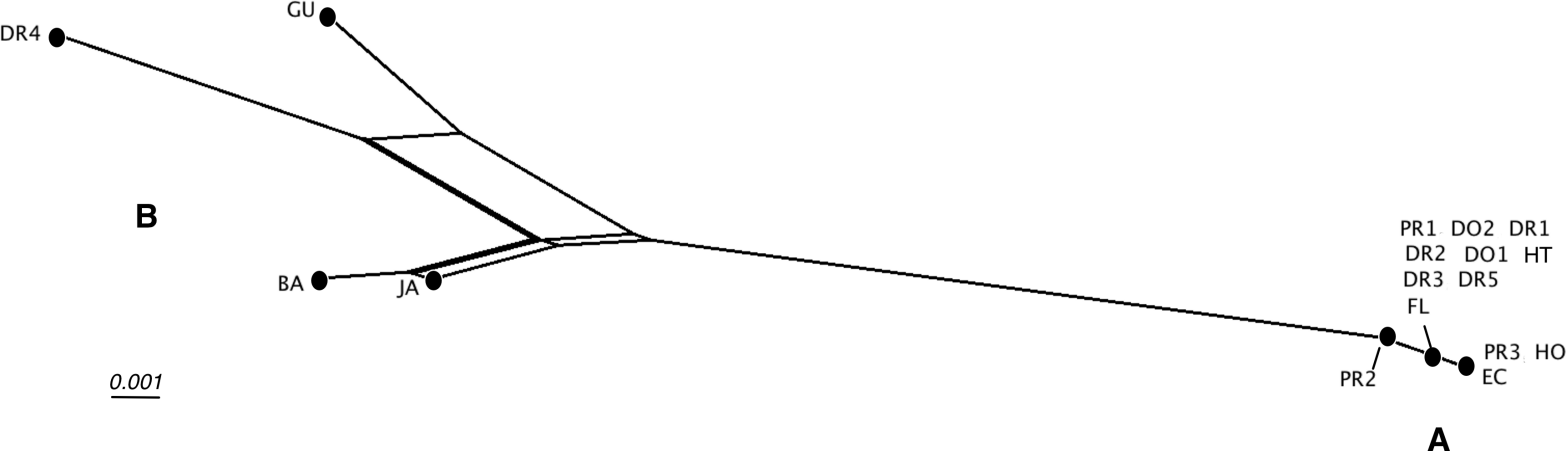
SplitsTree NeighborNet network of *Bulimulus guadalupensis* (Broderip, 1789), based on K2P distances and EqualAngle representation. Label explanation given in text.

Detailed analysis of the alignment shows that *B. guadalupensis* sequences from the following populations can be arranged into two groups of haplotypes (h): (Group A) (h1) DR1—Dominican Republic, La Cantera (USDA 110198); DR2—Dominican Republic, 4 km S Cabrera (USDA 110200); DR3—Dominican Republic, Come Pan (USDA 110197); DR5—Dominican Republic, Santo Domingo (RMNH 106983); PR1—Puerto Rico, Isla Vieques, Punta Arenas (USDA 110196); DO1—Dominica, Bellevue Chopin (USDA 100706); DO2—Dominica, Pointe Michel (USDA 100713); FL — USA, Florida, Miami, Coral Gables (USDA 110208), with the second haplotype (h2) differing in only one basepair at the following populations: PR2—Puerto Rico, Isla Vieques, Florida (USDA 110199). A third haplotype (h3) links the populations at PR3—Puerto Rico, Guayabo (USDA 110195) and those in EC—Ecuador (USDA 110210) and HO—Honduras (USDA 110162); there is a two basepair difference with h2. Group B contains the following populations: (h4) GU—Guadeloupe, Saint-Anne (USDA 110209); (h5) DR4—Dominican Republic, Las Terrenas (USDA 110201, 11 bp differences with h4); (h6) BA—Barbados, Sandy Lane (USDA 110207, 15 bp differences with h5, 13 bp with h4). The sequence from JA—Jamaica, Lucea (USDA 110194), although incomplete, is likely nearly identical to those from Barbados. In SplitsTrees4 group A appears to be rather homogeneous, while group B is more variable (Fig. 3). This was reflected in the higher values for group B in the BGA, both in EDS and NEDGS (Table S4).

**Figure 4:**
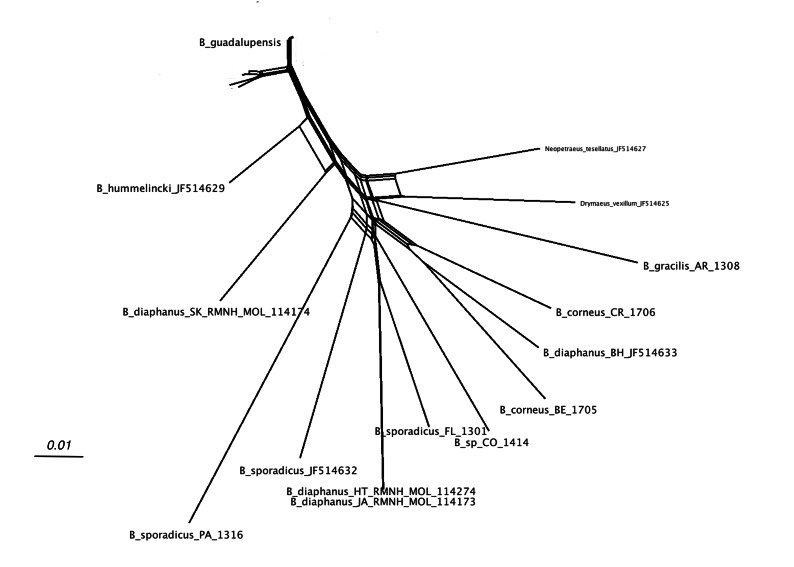
SplitsTree NeighborNet network of all *Bulimulus* species from the analysed dataset, based on K2P distances and EqualAngle representation. Label explanation given in text.

Figure 4 shows the network relations of the other species. *B. diaphanus* showed up in three different branches; the specimen from Nevis (RMNH.MOL.114174) was related to *B. hummelincki* (Breure, 1974) and corresponded to *B. diaphanus fraterculus* (Potiez & Michaud, 1835) (Breure, 1974). The specimens from Haiti (RMNH.MOL.114274) and Jamaica (RMNH.MOL.114173) were nearly identical, and correspond to *B. d. diaphanus* (Pfeiffer, 1855). The third sample from Bahamas (ANSP A22054) seemed to be more closely related to the Central American *B. corneus* (Sowerby I, 1833); this sample may have been misidentified. The three samples of *B. sporadicus* (d’Orbigny, 1835) appear to be partly unrelated; the specimens from Paraguay (RMNH.MOL.336661) and intercepted at Houston were on neighbouring branches (bootstrap 56; not shown). The sample from Florida (RMNH.MOL.336660), identified as the same species, was branching off *B. d. diaphanus* (bootstrap 86; not shown); this was corroborated in the MP tree, where the clade of *B. sporadicus* (Florida) and *B. diaphanus* (Haiti, Jamaica) had moderate support (bs: 72; not shown).

In the analyses using different species delimitation methods, the two groups within *B. guadalupensis* could be distinghuised using BGA with a 3% threshold, while at 4% threshold the difference was only marginal. Haplotype h4 showed a maximum distance of 0.041 ± 0.008. In the SDG method using ML the B group had a relatively high intraspecific/interspecific ratio, but a relatively low P ID(strict) and non-significant values of Rosenberg’s P_AB_ and Rodrigo’s P(RD); however, using BI the two groups were significantly different using the latter parameter, although the other parameters hardly differed (Table 2, Table S3). The ABGD method resulted in one group for the recursive partition with a prior of 0.1; 13 with 0.060; 14 with 0.036; 17 with 0.022, 0.013, 0.008, 0.005, 0.003, and 0.002; 19 groups were found with prior 0.001. The initial partition was stable on 17 groups at prior values of 0.022 and below. Of the two *B. guadalupensis* groups only group A was recognised with this method (Table 2, Table S4 ). On the contrary, in the GSI method both group A and B had a value of 1.00 (monophyly) with both ML and BI; all values were statistically significant, thus GSI suggested two species. Also within *B. diaphanus* two groups were suggested, but only one of these groups (from Jamaica and Haiti) had a significant value of 1.00. The analysed samples of *B. sporadicus* proved not to be monophyletic (Table 2, Table S5).

**Table 2:** Number of putative species delimited by different methods applied to the dataset. d, *Bulimulus diaphanus*; g, *B. guadalupensis*; s, *B. sporadicus*; BGA, Barcode gap analysis; SDG, species delimiting in Geneious; ABGD, Automated barcode gap discovery; GSI, Genealogical sorting index.

Putative species	BGA		SDG		ABGD	GSI	
	3%	4%	ML	BI		ML	BI
gA	+	−	+	+	+	+	+
gB	+	−	−	+	−	+	+
d1	+	+	−	+	+	+	+
d2	+	+	nc	nc	+	nc	nc
d3	+	+	nc	nc	+	nc	nc
s1	+	+	nc	nc	+	nc	nc
s2	+	+	nc	nc	+	nc	nc
s3	+	+	nc	nc	+	nc	nc

## Discussion

*Bulimulus guadalupensis* is known to be very variable morphologically, although measurements on samples throughout its distribution area led Breure (1974) conclude that “no infraspecific differentiation occurs.” The results on this species reported in the present paper are not univocal, the two groups that are discernable depend on the species delimitation method chosen. Thus drawing nomenclatural conclusions would—in the absence of clear external morphological differences—hinge totally on ambiguous molecular data. Since the locality data are often also not informative (e.g., in case of introduction or interception), no nomenclatural conclusions can be drawn.

For *B. diaphanus*, Breure (1974) recognised two subspecies, of which the samples studied in this paper from Haiti and Jamaica may be assigned to the nominate taxon, and the Nevis sample to *B. diaphanus fraterculus* (Potiez & Michaud, 1835) (Fig. 6B); more fresh tissue samples from different localities within its distribution range are needed to confirm the phylogenetic basis for the morphological differences reported earlier by Breure (1974). The polyphyly of samples of *B. diaphanus* in this study is possibly an indication of the presence of cryptic species in this group, but also here lack of data prevent me from drawing nomenclatoral conclusions.

**Figure 5:**
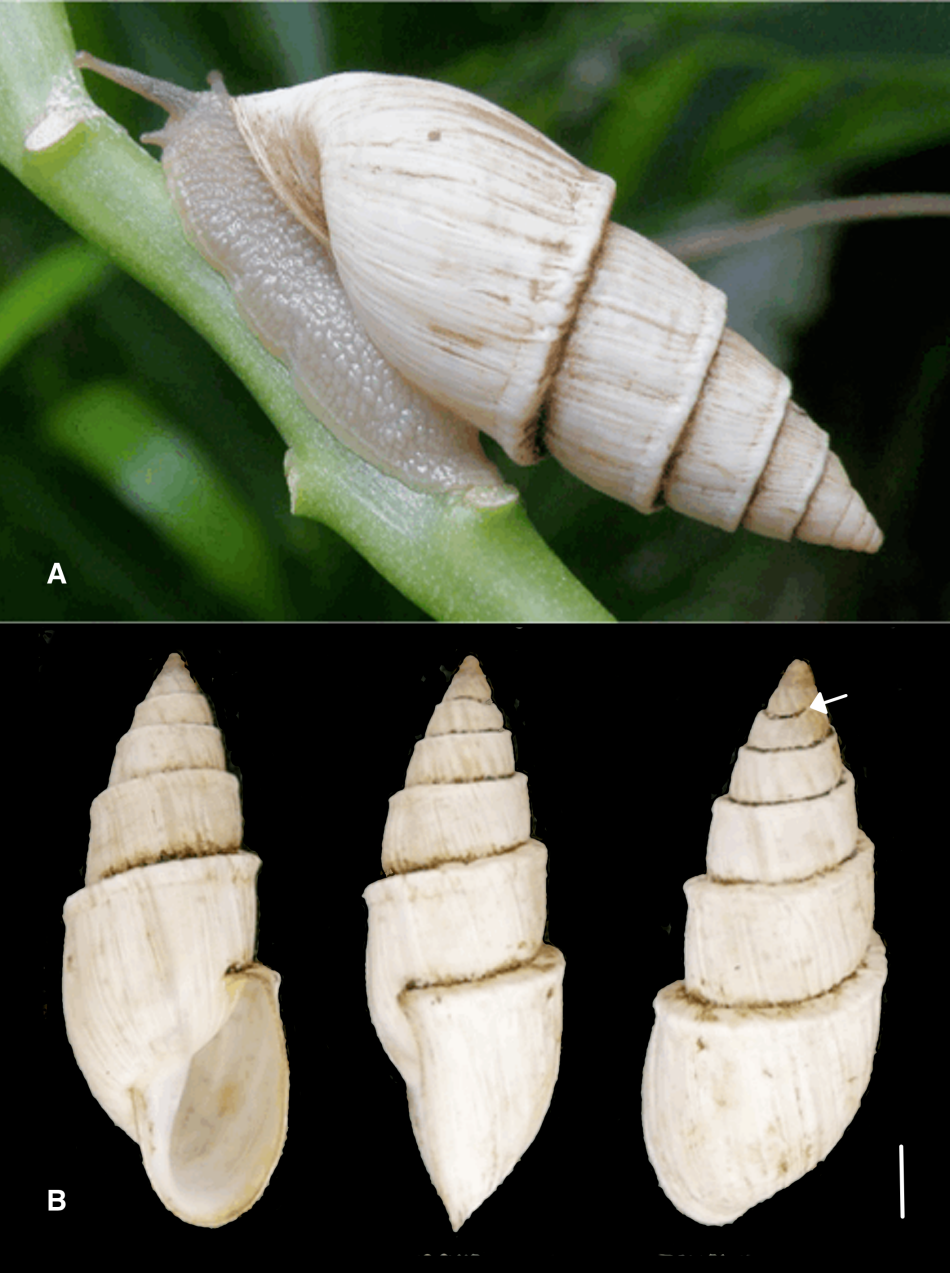
*Bulimulus sporadicus* (d’Orbigny, 1835), Paraguay, Concepción. U. Drechsel leg. RMNH.MOL.336661 (shell height 31.3 mm). (A) living specimen (photo by courtesy U. Drechsel). (B) different views of the shell, arrow indicating the place where the carination starts. Scale 5 mm.

**Figure 6:**
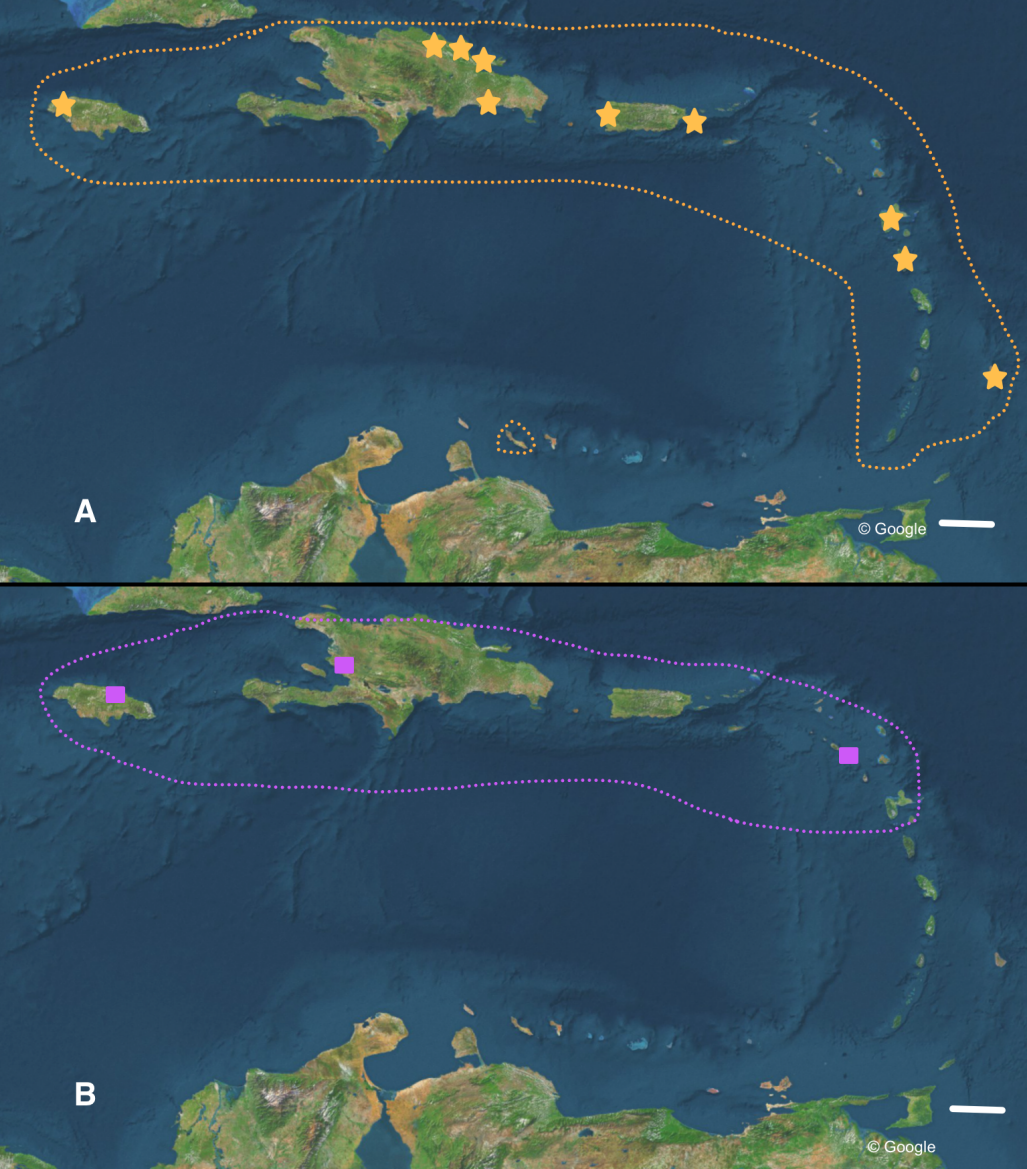
Distribution of (A) *Bulimulus guadalupensis* (orange), and (B) *B. diaphanus* (purple) in the West Indies. Dotted lines denote generalised distribution area, solid symbols sampled localities. Note that the Haitian locality is only an approximation. Map data: Google. Scale 100 km.

The occurrence of alien species in Florida seems to follow a ‘burst-diminishing pattern’, also observed in other introduced species, from becoming very common after the introduction to diminishing to (nearly) extirpated after some time (B Frank, pers. comm., 2014). The Miami population of *B. guadalupensis* currently lasted at least ten years. The Jacksonville population of *B*. aff. *sporadicus* was only recently discovered and seems to be thriving, at least within the city limits, possibly resulting from the potential absence of natural enemies (such as *Euglandina* snails). Simberloff & Gibbons (2004) have suggested that population collapses of introduced species may simply be temporary lows during a more or less regular boom-and-burst cycle. The time span of such a process may vary from species to species due to specific conditions, and is as yet unknown for these *Bulimulus*. Other introductions of these species in the Caribbean are less well documented. In Curaçao (Breure, 1975) a population of *B. guadalupensis* is still present, but only in gardens in some suburban areas outside Willemstad (G Van Buurt, pers. comm., 2014). From the network analysis (Fig. 4) it could be deduced that the introductions in Ecuador and Honduras may be traced back to the population in Guayabo, Puerto Rico, which may be linked to—in this case—export activities at the industrial park where the collection was made (Cf. Cowie et al., 2008). However,*Bulimulus* species are not considered to be high-risk exotics (Cowie et al., 2009). The introduction in Jamaica of this species may be linked to a population on a different island, and in this case molecular evidence suggests it may be traced to Barbados (see also Fig. 6A).

**Figure 7:**
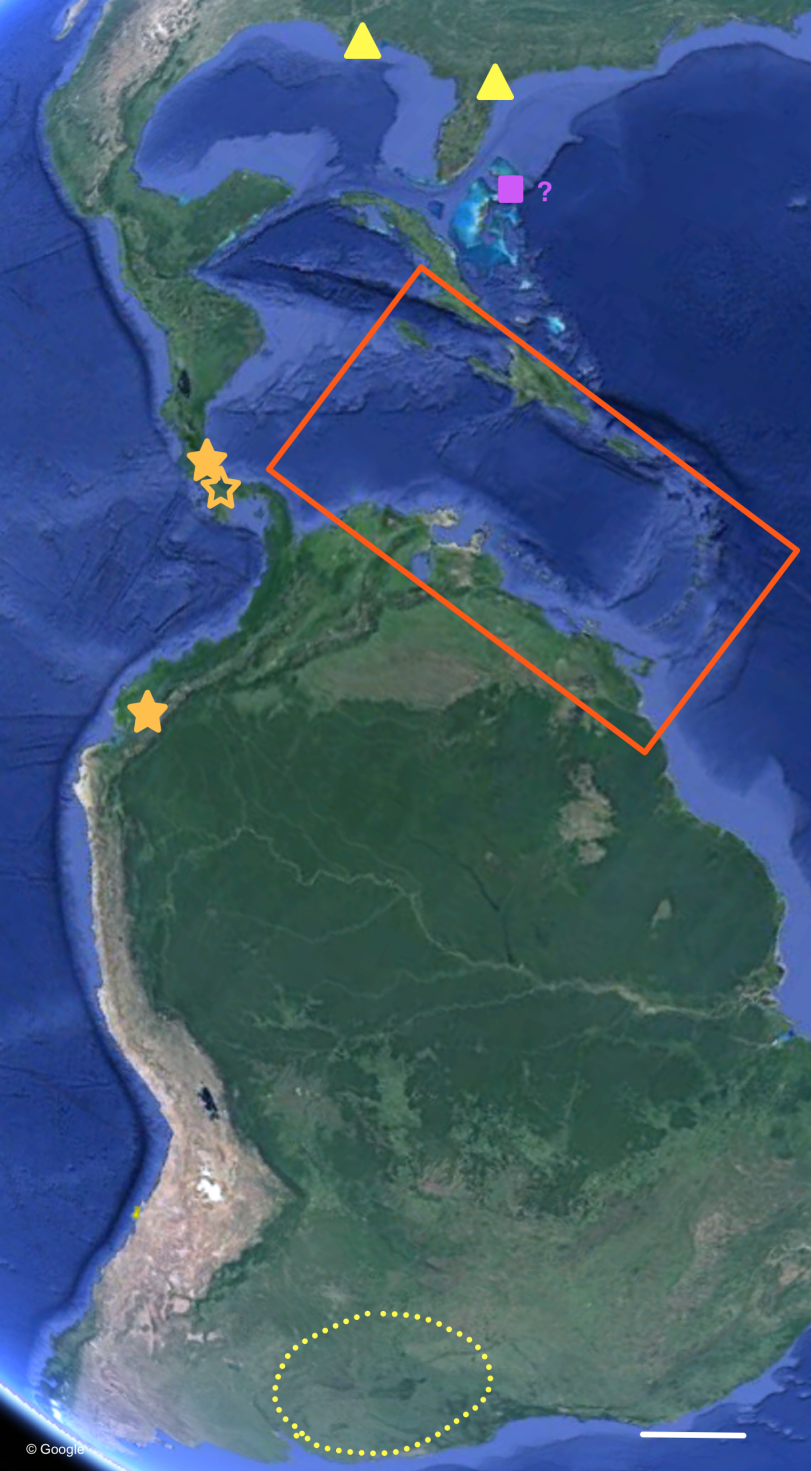
Distribution of *Bulimulus sporadicus* (yellow), *B. guadalupensis*, and *B. diaphanus* in the Neotropics. Red square is area enlarged in Fig. 6. Open symbol is a locality not sampled; other symbols, see legends of Fig. 6. Map Data: Google. Scale 500 km.

The phylogenetic analyses also provide some insights in the populations identified as *B. sporadicus* (d’Orbigny, 1835). It may be noted that only one specimen is from a natural population, viz. Paraguay, Concepción, albeit a somewhat aberrant specimen through damage of the shell at a juvenile stage (Fig. 5). The two other populations sampled are alien in the USA (Houston, intercepted by USDA; Jacksonville). This species is widespread in its area of origin and has been recorded from Bolivia, Brazil (Mato Grosso, Rio Grande do Sul, Santa Catarina), Paraguay, and Uruguay (Agudo-Padrón, 2014; Agudo-Padrón et al., 2014; Simone, 2006; see also Fig. 7). In Uruguay and Argentina (Provs. Buenos Aires, Chaco, Cordoba, Corrientes, Entre Rios, Formosa, Missiones, Tucumán) this taxon is replaced by the very similar *B. bonariensis* (Rafinesque, 1833) (Cuezzo, Miranda & Constanza Ovando, 2013; Scarabino, 2003). Miquel (1991) and Cuezzo, Miranda & Constanza Ovando (2013) consider these taxa as synonyms, with Rafinesque’s name having priority. However, the intraspecific variation within the large distribution range of this species makes it likely to be a species complex. Thus it may warrant a more detailed study, including molecular research, to clarify the possible existence of closely related taxa which may be difficult to distinguish on the basis of shell morphology alone.

This study shows that use of different species delimiting methods may produce different species hypotheses, and are thus are of limited value to arrive at an unequivocal taxonomic interpretation of these *Bulimulus* species, as was also observed by Prévot et al. (2013). In this case the relatively low number of samples analysed, and the use of only one genetic marker, makes it hard to convert the results into solid taxonomic decisions. Therefore it is suggested that the application of species delimitation methods may be of limited value as such, and should be complemented by other evidence, e.g., morphological studies.

Finally, the present study sheds some light on the usefulness of the barcoding method for rapid identification of intercepted snails of this group. Although the results strongly suggests that some of the physical movements (i.e., introduction as alien species in a distinct country) can be traced back to a source within the known distribution area, it is also clear that this holds true for the better known species only (e.g., *B. guadalupensis*). For others, especially for taxa that are morphologically very similar, the extent and reliability of the current database in GenBank is insufficient, especially with respect to areas where *Bulimulus* species are known to be native. In a wider perspective, where the effects of the global economy on non-marine gastropod introductions are becoming more and more manifest (Robinson, 1999), this is a problematic conclusion.

## Supplemental Information

10.7717/peerj.1836/supp-1 Figure S1 Neighbour-joining phylogeny for *Bulimulus* species, based on 654bp cytochrome oxidase I mitochondrila DNA. Bootstrap values of 70 and above are presented to the left of the nodes indicated by black dots. Scale bar in substitutions/site

10.7717/peerj.1836/supp-2 Figure S2 Maximum-parsimony phylogeny for *Bulimulus* species, based on 654bp cytochrome oxidase I mitochondrila DNA. Bootstrap values of 90 and above are presented to the left of the nodes indicated by black dots

10.7717/peerj.1836/supp-3 Table S1 Delimitation of MOTUs using K2P distances and standard error at 3% threshold. gA and gB, *Bulimulus guadalupensis* group A respectively B

10.7717/peerj.1836/supp-4 Table S2 Delimitation of MOTUs using K2P distances and standard error at 4% threshold. gA and gB, *Bulimulus guadalupensis* group A respectively B

10.7717/peerj.1836/supp-5 Table S3 Species delimiting as implemented in Geneious, using ML and BI for both the total dataset and a subgroup of MOTUs. Closest species, Intraspecific distance, Interspecies distance, ratio of Intra/Interspecific, P ID(strict), Rosenberg’s Pab, and Rodrigo’s P(RD) are indicated. Colours code for significance. c, d, g, gr, hu, s, and sp. correspond with the respective taxon names; NA, not applicable

10.7717/peerj.1836/supp-6 Table S4 Mean p-distances between and within (diagonal) the different MOTUs based on the dataset analysed. EDS, Estimates of Evolutionary Divergence between Sequences; NEDGS, Net Evolutionary Divergence between Group of Sequences. c, d, g, gr, hu, s, and sp. correspond with the respective taxon names; n/c, not calculated. Colours code the corresponding groups

10.7717/peerj.1836/supp-7 Table S5 Results for different combinations of MOTUs, using rooted trees of both ML and BI analyses, in terms of genealogical sorting index and corresponding p-values based on a permutation test of 10,000 replicates. Colours code the corresponding groups

## Additional Information and Declarations

### DNA Deposition

The following information was supplied regarding the deposition of DNA sequences: GenBank accession numbers can be found in Table 1.

### Data Availability

The following information was supplied regarding data availability: All raw data are either published (GenBank) or provided as supplementary files.
