# Supplementary figures and images for: Caribbean Bulimulus revisited: physical moves and molecular traces (Mollusca, Gastropoda, Bulimulidae)
Source: PeerJ. 2016 Mar 29;4:e1836. doi: 10.7717/peerj.1836 (PMC4824910; doi:10.7717/peerj.1836)

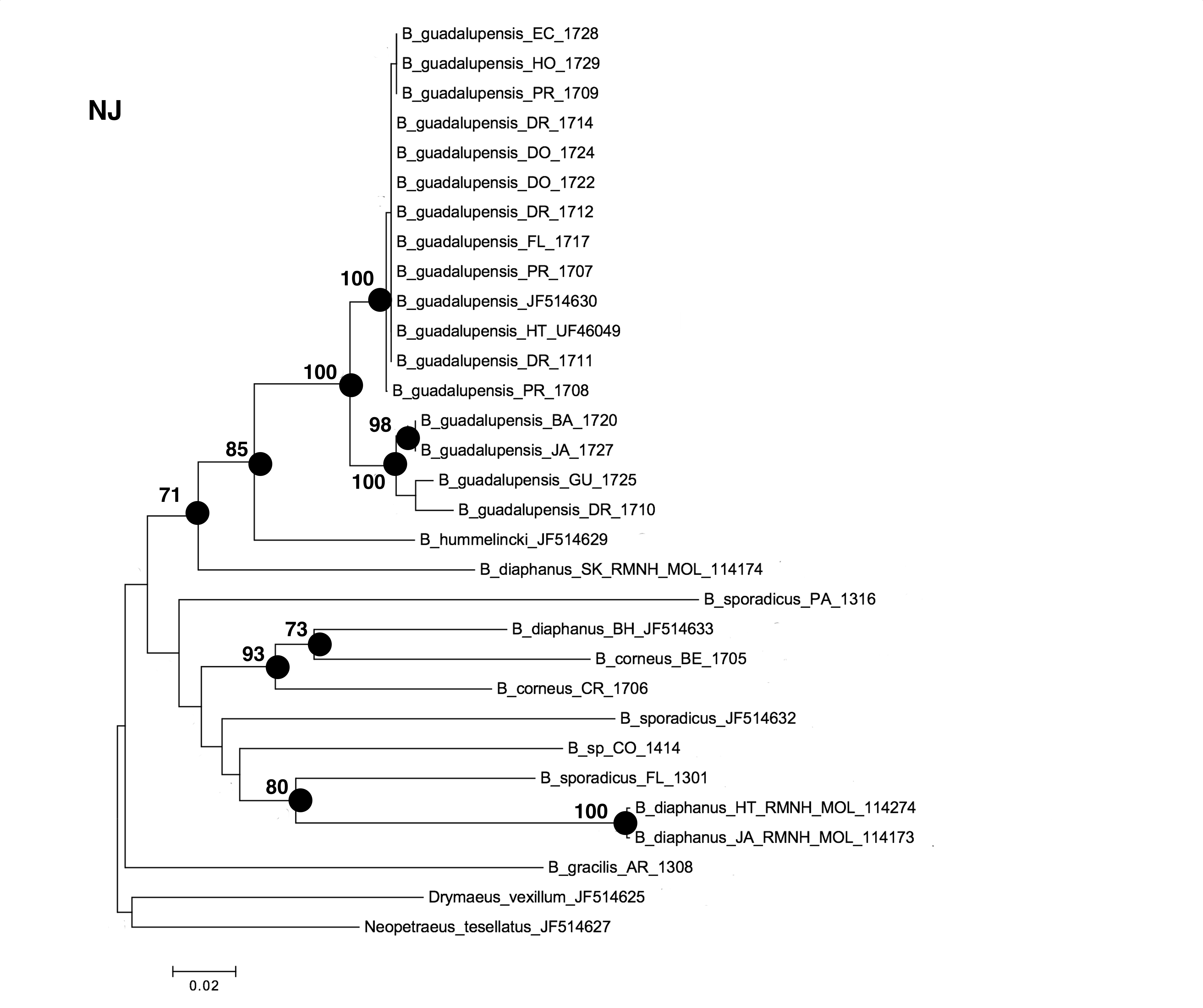

Supplement: Figure S1 [file peerj-04-1836-s001.png]

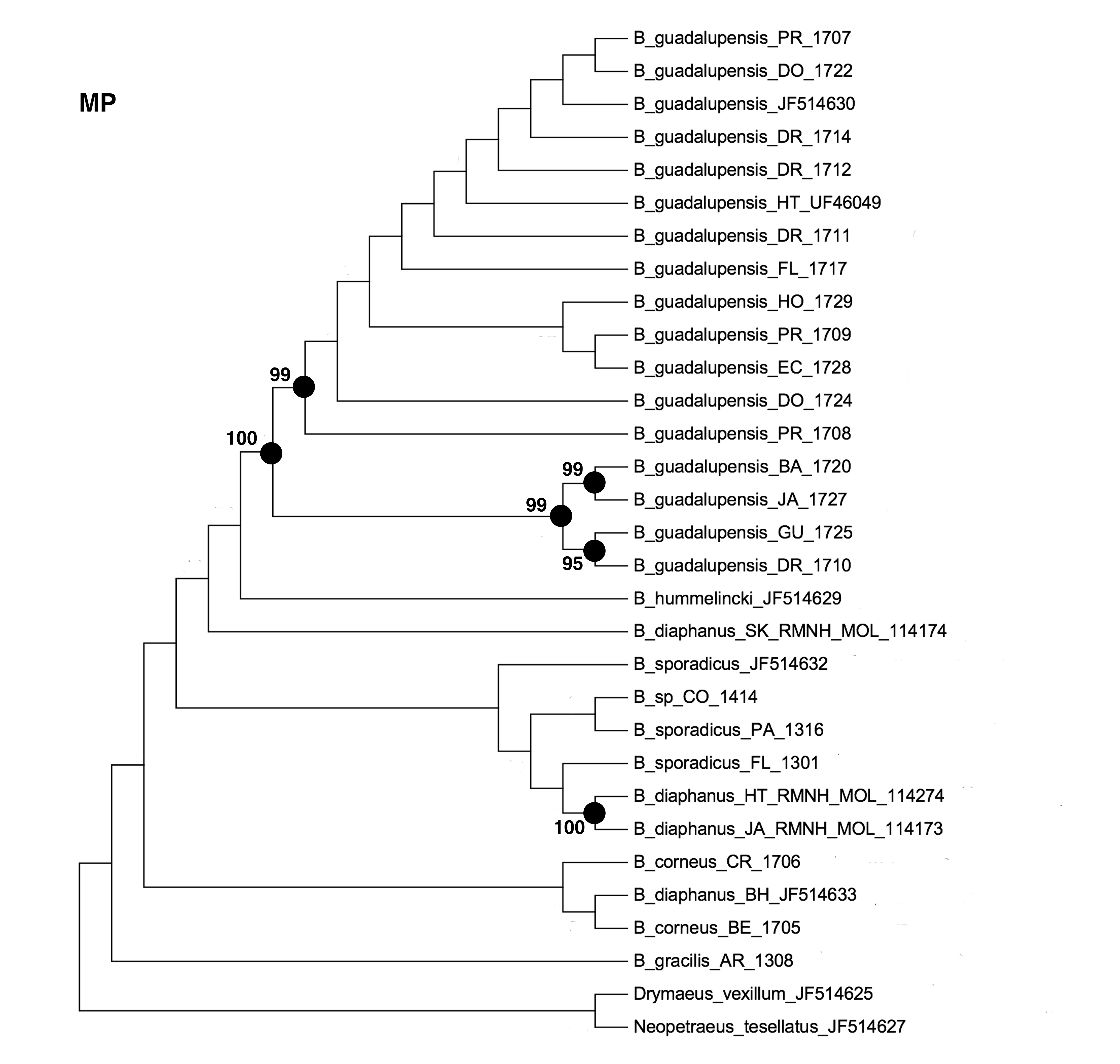

Supplement: Figure S2 [file peerj-04-1836-s002.png]
