# Supplementary material for: Caribbean Bulimulus revisited: physical moves and molecular traces (Mollusca, Gastropoda, Bulimulidae)
Source: PeerJ. 2016 Mar 29;4:e1836. doi: 10.7717/peerj.1836 (PMC4824910; doi:10.7717/peerj.1836)
Supplement: Table S2 [file peerj-04-1836-s004.pdf]

Species 1	Species 2	Dist	Std. Err	group
Drymaeus_vexillum_JF514625	Neopetraeus_tesellatus_JF514627	0.165	0.018	
Drymaeus_vexillum_JF514625	B_hummelincki_JF514629	0.171	0.018	
Neopetraeus_tesellatus_JF514627	B_hummelincki_JF514629	0.207	0.022	
Drymaeus_vexillum_JF514625	B_diaphanus_BH_JF514633	0.209	0.022	
Neopetraeus_tesellatus_JF514627	B_diaphanus_BH_JF514633	0.208	0.022	
B_hummelincki_JF514629	B_diaphanus_BH_JF514633	0.212	0.022	
Drymaeus_vexillum_JF514625	B_diaphanus_HT_RMNH_MOL_114274	0.262	0.024	
Neopetraeus_tesellatus_JF514627	B_diaphanus_HT_RMNH_MOL_114274	0.219	0.022	
B_hummelincki_JF514629	B_diaphanus_HT_RMNH_MOL_114274	0.255	0.024	
B_diaphanus_BH_JF514633	B_diaphanus_HT_RMNH_MOL_114274	0.227	0.023	
Drymaeus_vexillum_JF514625	B_diaphanus_JA_RMNH_MOL_114173	0.261	0.024	
Neopetraeus_tesellatus_JF514627	B_diaphanus_JA_RMNH_MOL_114173	0.219	0.022	
B_hummelincki_JF514629	B_diaphanus_JA_RMNH_MOL_114173	0.255	0.024	
B_diaphanus_BH_JF514633	B_diaphanus_JA_RMNH_MOL_114173	0.225	0.023	
B_diaphanus_HT_RMNH_MOL_114274	B_diaphanus_JA_RMNH_MOL_114173	0.002	0.002	
Drymaeus_vexillum_JF514625	B_diaphanus_SK_RMNH_MOL_114174	0.223	0.023	
Neopetraeus_tesellatus_JF514627	B_diaphanus_SK_RMNH_MOL_114174	0.212	0.022	
B_hummelincki_JF514629	B_diaphanus_SK_RMNH_MOL_114174	0.148	0.017	
B_diaphanus_BH_JF514633	B_diaphanus_SK_RMNH_MOL_114174	0.195	0.021	
B_diaphanus_HT_RMNH_MOL_114274	B_diaphanus_SK_RMNH_MOL_114174	0.258	0.024	
B_diaphanus_JA_RMNH_MOL_114173	B_diaphanus_SK_RMNH_MOL_114174	0.259	0.024	
Drymaeus_vexillum_JF514625	B_guadalupensis_HT_UF46049	0.186	0.019	
Neopetraeus_tesellatus_JF514627	B_guadalupensis_HT_UF46049	0.156	0.017	
B_hummelincki_JF514629	B_guadalupensis_HT_UF46049	0.094	0.013	
B_diaphanus_BH_JF514633	B_guadalupensis_HT_UF46049	0.187	0.020	
B_diaphanus_HT_RMNH_MOL_114274	B_guadalupensis_HT_UF46049	0.241	0.024	
B_diaphanus_JA_RMNH_MOL_114173	B_guadalupensis_HT_UF46049	0.241	0.024	
B_diaphanus_SK_RMNH_MOL_114174	B_guadalupensis_HT_UF46049	0.151	0.018	
Drymaeus_vexillum_JF514625	B_guadalupensis_JF514630	0.186	0.019	
Neopetraeus_tesellatus_JF514627	B_guadalupensis_JF514630	0.156	0.017	
B_hummelincki_JF514629	B_guadalupensis_JF514630	0.094	0.013	
B_diaphanus_BH_JF514633	B_guadalupensis_JF514630	0.187	0.020	
B_diaphanus_HT_RMNH_MOL_114274	B_guadalupensis_JF514630	0.241	0.024	
B_diaphanus_JA_RMNH_MOL_114173	B_guadalupensis_JF514630	0.241	0.024	
B_diaphanus_SK_RMNH_MOL_114174	B_guadalupensis_JF514630	0.151	0.018	
B_guadalupensis_HT_UF46049	B_guadalupensis_JF514630	-0.000	0.000	gA
Drymaeus_vexillum_JF514625	B_sporadicus_FL_1301	0.221	0.021	
Neopetraeus_tesellatus_JF514627	B_sporadicus_FL_1301	0.229	0.022	
B_hummelincki_JF514629	B_sporadicus_FL_1301	0.212	0.020	
B_diaphanus_BH_JF514633	B_sporadicus_FL_1301	0.179	0.020	
B_diaphanus_HT_RMNH_MOL_114274	B_sporadicus_FL_1301	0.183	0.020	
B_diaphanus_JA_RMNH_MOL_114173	B_sporadicus_FL_1301	0.181	0.020	
B_diaphanus_SK_RMNH_MOL_114174	B_sporadicus_FL_1301	0.223	0.022	
B_guadalupensis_HT_UF46049	B_sporadicus_FL_1301	0.203	0.021	

B_guadalupensis_JF514630	B_sporadicus_FL_1301	0.203	0.021	
Drymaeus_vexillum_JF514625	B_sp_CO_1414	0.196	0.020	
Neopetraeus_tesellatus_JF514627	B_sp_CO_1414	0.233	0.022	
B_hummelincki_JF514629	B_sp_CO_1414	0.210	0.021	
B_diaphanus_BH_JF514633	B_sp_CO_1414	0.231	0.024	
B_diaphanus_HT_RMNH_MOL_114274	B_sp_CO_1414	0.229	0.023	
B_diaphanus_JA_RMNH_MOL_114173	B_sp_CO_1414	0.229	0.023	
B_diaphanus_SK_RMNH_MOL_114174	B_sp_CO_1414	0.231	0.023	
B_guadalupensis_HT_UF46049	B_sp_CO_1414	0.212	0.021	
B_guadalupensis_JF514630	B_sp_CO_1414	0.212	0.021	
B_sporadicus_FL_1301	B_sp_CO_1414	0.195	0.019	
Drymaeus_vexillum_JF514625	B_gracilis_AR_1308	0.226	0.023	
Neopetraeus_tesellatus_JF514627	B_gracilis_AR_1308	0.219	0.021	
B_hummelincki_JF514629	B_gracilis_AR_1308	0.240	0.022	
B_diaphanus_BH_JF514633	B_gracilis_AR_1308	0.262	0.025	
B_diaphanus_HT_RMNH_MOL_114274	B_gracilis_AR_1308	0.275	0.024	
B_diaphanus_JA_RMNH_MOL_114173	B_gracilis_AR_1308	0.275	0.024	
B_diaphanus_SK_RMNH_MOL_114174	B_gracilis_AR_1308	0.215	0.021	
B_guadalupensis_HT_UF46049	B_gracilis_AR_1308	0.220	0.021	
B_guadalupensis_JF514630	B_gracilis_AR_1308	0.220	0.021	
B_sporadicus_FL_1301	B_gracilis_AR_1308	0.254	0.024	
B_sp_CO_1414	B_gracilis_AR_1308	0.272	0.025	
Drymaeus_vexillum_JF514625	B_sporadicus_PA_1316	0.297	0.026	
Neopetraeus_tesellatus_JF514627	B_sporadicus_PA_1316	0.307	0.028	
B_hummelincki_JF514629	B_sporadicus_PA_1316	0.268	0.025	
B_diaphanus_BH_JF514633	B_sporadicus_PA_1316	0.280	0.026	
B_diaphanus_HT_RMNH_MOL_114274	B_sporadicus_PA_1316	0.283	0.025	
B_diaphanus_JA_RMNH_MOL_114173	B_sporadicus_PA_1316	0.283	0.025	
B_diaphanus_SK_RMNH_MOL_114174	B_sporadicus_PA_1316	0.310	0.028	
B_guadalupensis_HT_UF46049	B_sporadicus_PA_1316	0.251	0.023	
B_guadalupensis_JF514630	B_sporadicus_PA_1316	0.251	0.023	
B_sporadicus_FL_1301	B_sporadicus_PA_1316	0.274	0.026	
B_sp_CO_1414	B_sporadicus_PA_1316	0.276	0.025	
B_gracilis_AR_1308	B_sporadicus_PA_1316	0.311	0.026	
Drymaeus_vexillum_JF514625	B_guadalupensis_PR_1707	0.186	0.019	
Neopetraeus_tesellatus_JF514627	B_guadalupensis_PR_1707	0.156	0.017	
B_hummelincki_JF514629	B_guadalupensis_PR_1707	0.094	0.013	
B_diaphanus_BH_JF514633	B_guadalupensis_PR_1707	0.187	0.020	
B_diaphanus_HT_RMNH_MOL_114274	B_guadalupensis_PR_1707	0.241	0.024	
B_diaphanus_JA_RMNH_MOL_114173	B_guadalupensis_PR_1707	0.241	0.024	
B_diaphanus_SK_RMNH_MOL_114174	B_guadalupensis_PR_1707	0.151	0.018	
B_guadalupensis_HT_UF46049	B_guadalupensis_PR_1707	-0.000	0.000	gA
B_guadalupensis_JF514630	B_guadalupensis_PR_1707	-0.000	0.000	gA
B_sporadicus_FL_1301	B_guadalupensis_PR_1707	0.203	0.021	
B_sp_CO_1414	B_guadalupensis_PR_1707	0.212	0.021	

B_gracilis_AR_1308	B_guadalupensis_PR_1707	0.220	0.021	
B_sporadicus_PA_1316	B_guadalupensis_PR_1707	0.251	0.023	
Drymaeus_vexillum_JF514625	B_guadalupensis_FL_1717	0.186	0.019	
Neopetraeus_tesellatus_JF514627	B_guadalupensis_FL_1717	0.156	0.017	
B_hummelincki_JF514629	B_guadalupensis_FL_1717	0.094	0.013	
B_diaphanus_BH_JF514633	B_guadalupensis_FL_1717	0.187	0.020	
B_diaphanus_HT_RMNH_MOL_114274	B_guadalupensis_FL_1717	0.241	0.024	
B_diaphanus_JA_RMNH_MOL_114173	B_guadalupensis_FL_1717	0.241	0.024	
B_diaphanus_SK_RMNH_MOL_114174	B_guadalupensis_FL_1717	0.151	0.018	
B_guadalupensis_HT_UF46049	B_guadalupensis_FL_1717	-0.000	0.000	gA
B_guadalupensis_JF514630	B_guadalupensis_FL_1717	-0.000	0.000	gA
B_sporadicus_FL_1301	B_guadalupensis_FL_1717	0.203	0.021	
B_sp_CO_1414	B_guadalupensis_FL_1717	0.212	0.021	
B_gracilis_AR_1308	B_guadalupensis_FL_1717	0.220	0.021	
B_sporadicus_PA_1316	B_guadalupensis_FL_1717	0.251	0.023	
B_guadalupensis_PR_1707	B_guadalupensis_FL_1717	-0.000	0.000	gA
Drymaeus_vexillum_JF514625	B_guadalupensis_DR_1712	0.186	0.019	
Neopetraeus_tesellatus_JF514627	B_guadalupensis_DR_1712	0.156	0.017	
B_hummelincki_JF514629	B_guadalupensis_DR_1712	0.094	0.013	
B_diaphanus_BH_JF514633	B_guadalupensis_DR_1712	0.187	0.020	
B_diaphanus_HT_RMNH_MOL_114274	B_guadalupensis_DR_1712	0.241	0.024	
B_diaphanus_JA_RMNH_MOL_114173	B_guadalupensis_DR_1712	0.241	0.024	
B_diaphanus_SK_RMNH_MOL_114174	B_guadalupensis_DR_1712	0.151	0.018	
B_guadalupensis_HT_UF46049	B_guadalupensis_DR_1712	-0.000	0.000	gA
B_guadalupensis_JF514630	B_guadalupensis_DR_1712	-0.000	0.000	gA
B_sporadicus_FL_1301	B_guadalupensis_DR_1712	0.203	0.021	
B_sp_CO_1414	B_guadalupensis_DR_1712	0.212	0.021	
B_gracilis_AR_1308	B_guadalupensis_DR_1712	0.220	0.021	
B_sporadicus_PA_1316	B_guadalupensis_DR_1712	0.251	0.023	
B_guadalupensis_PR_1707	B_guadalupensis_DR_1712	-0.000	0.000	
B_guadalupensis_FL_1717	B_guadalupensis_DR_1712	-0.000	0.000	
Drymaeus_vexillum_JF514625	B_guadalupensis_DO_1722	0.186	0.019	
Neopetraeus_tesellatus_JF514627	B_guadalupensis_DO_1722	0.156	0.017	
B_hummelincki_JF514629	B_guadalupensis_DO_1722	0.094	0.013	
B_diaphanus_BH_JF514633	B_guadalupensis_DO_1722	0.187	0.020	
B_diaphanus_HT_RMNH_MOL_114274	B_guadalupensis_DO_1722	0.241	0.024	
B_diaphanus_JA_RMNH_MOL_114173	B_guadalupensis_DO_1722	0.241	0.024	
B_diaphanus_SK_RMNH_MOL_114174	B_guadalupensis_DO_1722	0.151	0.018	
B_guadalupensis_HT_UF46049	B_guadalupensis_DO_1722	-0.000	0.000	gA
B_guadalupensis_JF514630	B_guadalupensis_DO_1722	-0.000	0.000	gA
B_sporadicus_FL_1301	B_guadalupensis_DO_1722	0.203	0.021	
B_sp_CO_1414	B_guadalupensis_DO_1722	0.212	0.021	
B_gracilis_AR_1308	B_guadalupensis_DO_1722	0.220	0.021	
B_sporadicus_PA_1316	B_guadalupensis_DO_1722	0.251	0.023	
B_guadalupensis_PR_1707	B_guadalupensis_DO_1722	-0.000	0.000	gA

B_guadalupensis_FL_1717	B_guadalupensis_DO_1722	-0.000	0.000	gA
B_guadalupensis_DR_1712	B_guadalupensis_DO_1722	-0.000	0.000	gA
Drymaeus_vexillum_JF514625	B_guadalupensis_DO_1724	0.186	0.019	
Neopetraeus_tesellatus_JF514627	B_guadalupensis_DO_1724	0.156	0.017	
B_hummelincki_JF514629	B_guadalupensis_DO_1724	0.094	0.013	
B_diaphanus_BH_JF514633	B_guadalupensis_DO_1724	0.187	0.020	
B_diaphanus_HT_RMNH_MOL_114274	B_guadalupensis_DO_1724	0.241	0.024	
B_diaphanus_JA_RMNH_MOL_114173	B_guadalupensis_DO_1724	0.241	0.024	
B_diaphanus_SK_RMNH_MOL_114174	B_guadalupensis_DO_1724	0.151	0.018	
B_guadalupensis_HT_UF46049	B_guadalupensis_DO_1724	-0.000	0.000	gA
B_guadalupensis_JF514630	B_guadalupensis_DO_1724	-0.000	0.000	gA
B_sporadicus_FL_1301	B_guadalupensis_DO_1724	0.203	0.021	
B_sp_CO_1414	B_guadalupensis_DO_1724	0.212	0.021	
B_gracilis_AR_1308	B_guadalupensis_DO_1724	0.220	0.021	
B_sporadicus_PA_1316	B_guadalupensis_DO_1724	0.251	0.023	
B_guadalupensis_PR_1707	B_guadalupensis_DO_1724	-0.000	0.000	gA
B_guadalupensis_FL_1717	B_guadalupensis_DO_1724	-0.000	0.000	gA
B_guadalupensis_DR_1712	B_guadalupensis_DO_1724	-0.000	0.000	gA
B_guadalupensis_DO_1722	B_guadalupensis_DO_1724	-0.000	0.000	gA
Drymaeus_vexillum_JF514625	B_guadalupensis_DR_1711	0.186	0.019	
Neopetraeus_tesellatus_JF514627	B_guadalupensis_DR_1711	0.156	0.017	
B_hummelincki_JF514629	B_guadalupensis_DR_1711	0.094	0.013	
B_diaphanus_BH_JF514633	B_guadalupensis_DR_1711	0.187	0.020	
B_diaphanus_HT_RMNH_MOL_114274	B_guadalupensis_DR_1711	0.241	0.024	
B_diaphanus_JA_RMNH_MOL_114173	B_guadalupensis_DR_1711	0.241	0.024	
B_diaphanus_SK_RMNH_MOL_114174	B_guadalupensis_DR_1711	0.151	0.018	
B_guadalupensis_HT_UF46049	B_guadalupensis_DR_1711	-0.000	0.000	gA
B_guadalupensis_JF514630	B_guadalupensis_DR_1711	-0.000	0.000	gA
B_sporadicus_FL_1301	B_guadalupensis_DR_1711	0.203	0.021	
B_sp_CO_1414	B_guadalupensis_DR_1711	0.212	0.021	
B_gracilis_AR_1308	B_guadalupensis_DR_1711	0.220	0.021	
B_sporadicus_PA_1316	B_guadalupensis_DR_1711	0.251	0.023	
B_guadalupensis_PR_1707	B_guadalupensis_DR_1711	-0.000	0.000	gA
B_guadalupensis_FL_1717	B_guadalupensis_DR_1711	-0.000	0.000	gA
B_guadalupensis_DR_1712	B_guadalupensis_DR_1711	-0.000	0.000	gA
B_guadalupensis_DO_1722	B_guadalupensis_DR_1711	-0.000	0.000	gA
B_guadalupensis_DO_1724	B_guadalupensis_DR_1711	-0.000	0.000	gA
Drymaeus_vexillum_JF514625	B_guadalupensis_DR_1714	0.186	0.019	
Neopetraeus_tesellatus_JF514627	B_guadalupensis_DR_1714	0.156	0.017	
B_hummelincki_JF514629	B_guadalupensis_DR_1714	0.094	0.013	
B_diaphanus_BH_JF514633	B_guadalupensis_DR_1714	0.187	0.020	
B_diaphanus_HT_RMNH_MOL_114274	B_guadalupensis_DR_1714	0.241	0.024	
B_diaphanus_JA_RMNH_MOL_114173	B_guadalupensis_DR_1714	0.241	0.024	
B_diaphanus_SK_RMNH_MOL_114174	B_guadalupensis_DR_1714	0.151	0.018	
B_guadalupensis_HT_UF46049	B_guadalupensis_DR_1714	-0.000	0.000	gA

B_guadalupensis_JF514630	B_guadalupensis_DR_1714	-0.000	0.000	gA
B_sporadicus_FL_1301	B_guadalupensis_DR_1714	0.203	0.021	
B_sp_CO_1414	B_guadalupensis_DR_1714	0.212	0.021	
B_gracilis_AR_1308	B_guadalupensis_DR_1714	0.220	0.021	
B_sporadicus_PA_1316	B_guadalupensis_DR_1714	0.251	0.023	
B_guadalupensis_PR_1707	B_guadalupensis_DR_1714	-0.000	0.000	gA
B_guadalupensis_FL_1717	B_guadalupensis_DR_1714	-0.000	0.000	gA
B_guadalupensis_DR_1712	B_guadalupensis_DR_1714	-0.000	0.000	gA
B_guadalupensis_DO_1722	B_guadalupensis_DR_1714	-0.000	0.000	gA
B_guadalupensis_DO_1724	B_guadalupensis_DR_1714	-0.000	0.000	gA
B_guadalupensis_DR_1711	B_guadalupensis_DR_1714	-0.000	0.000	gA
Drymaeus_vexillum_JF514625	B_guadalupensis_PR_1709	0.189	0.020	
Neopetraeus_tesellatus_JF514627	B_guadalupensis_PR_1709	0.158	0.017	
B_hummelincki_JF514629	B_guadalupensis_PR_1709	0.096	0.013	
B_diaphanus_BH_JF514633	B_guadalupensis_PR_1709	0.187	0.020	
B_diaphanus_HT_RMNH_MOL_114274	B_guadalupensis_PR_1709	0.241	0.024	
B_diaphanus_JA_RMNH_MOL_114173	B_guadalupensis_PR_1709	0.241	0.024	
B_diaphanus_SK_RMNH_MOL_114174	B_guadalupensis_PR_1709	0.153	0.018	
B_guadalupensis_HT_UF46049	B_guadalupensis_PR_1709	0.002	0.002	gA
B_guadalupensis_JF514630	B_guadalupensis_PR_1709	0.002	0.002	gA
B_sporadicus_FL_1301	B_guadalupensis_PR_1709	0.203	0.021	
B_sp_CO_1414	B_guadalupensis_PR_1709	0.214	0.021	
B_gracilis_AR_1308	B_guadalupensis_PR_1709	0.220	0.021	
B_sporadicus_PA_1316	B_guadalupensis_PR_1709	0.251	0.023	
B_guadalupensis_PR_1707	B_guadalupensis_PR_1709	0.002	0.002	gA
B_guadalupensis_FL_1717	B_guadalupensis_PR_1709	0.002	0.002	gA
B_guadalupensis_DR_1712	B_guadalupensis_PR_1709	0.002	0.002	gA
B_guadalupensis_DO_1722	B_guadalupensis_PR_1709	0.002	0.002	gA
B_guadalupensis_DO_1724	B_guadalupensis_PR_1709	0.002	0.002	gA
B_guadalupensis_DR_1711	B_guadalupensis_PR_1709	0.002	0.002	gA
B_guadalupensis_DR_1714	B_guadalupensis_PR_1709	0.002	0.002	gA
Drymaeus_vexillum_JF514625	B_guadalupensis_EC_1728	0.189	0.020	
Neopetraeus_tesellatus_JF514627	B_guadalupensis_EC_1728	0.158	0.017	
B_hummelincki_JF514629	B_guadalupensis_EC_1728	0.096	0.013	
B_diaphanus_BH_JF514633	B_guadalupensis_EC_1728	0.187	0.020	
B_diaphanus_HT_RMNH_MOL_114274	B_guadalupensis_EC_1728	0.241	0.024	
B_diaphanus_JA_RMNH_MOL_114173	B_guadalupensis_EC_1728	0.241	0.024	
B_diaphanus_SK_RMNH_MOL_114174	B_guadalupensis_EC_1728	0.153	0.018	
B_guadalupensis_HT_UF46049	B_guadalupensis_EC_1728	0.002	0.002	gA
B_guadalupensis_JF514630	B_guadalupensis_EC_1728	0.002	0.002	gA
B_sporadicus_FL_1301	B_guadalupensis_EC_1728	0.203	0.021	
B_sp_CO_1414	B_guadalupensis_EC_1728	0.214	0.021	
B_gracilis_AR_1308	B_guadalupensis_EC_1728	0.220	0.021	
B_sporadicus_PA_1316	B_guadalupensis_EC_1728	0.251	0.023	
B_guadalupensis_PR_1707	B_guadalupensis_EC_1728	0.002	0.002	gA

B_guadalupensis_FL_1717	B_guadalupensis_EC_1728	0.002	0.002	gA
B_guadalupensis_DR_1712	B_guadalupensis_EC_1728	0.002	0.002	gA
B_guadalupensis_DO_1722	B_guadalupensis_EC_1728	0.002	0.002	gA
B_guadalupensis_DO_1724	B_guadalupensis_EC_1728	0.002	0.002	gA
B_guadalupensis_DR_1711	B_guadalupensis_EC_1728	0.002	0.002	gA
B_guadalupensis_DR_1714	B_guadalupensis_EC_1728	0.002	0.002	gA
B_guadalupensis_PR_1709	B_guadalupensis_EC_1728	-0.000	0.000	gA
Drymaeus_vexillum_JF514625	B_guadalupensis_HO_1729	0.189	0.020	
Neopetraeus_tesellatus_JF514627	B_guadalupensis_HO_1729	0.158	0.017	
B_hummelincki_JF514629	B_guadalupensis_HO_1729	0.096	0.013	
B_diaphanus_BH_JF514633	B_guadalupensis_HO_1729	0.187	0.020	
B_diaphanus_HT_RMNH_MOL_114274	B_guadalupensis_HO_1729	0.241	0.024	
B_diaphanus_JA_RMNH_MOL_114173	B_guadalupensis_HO_1729	0.241	0.024	
B_diaphanus_SK_RMNH_MOL_114174	B_guadalupensis_HO_1729	0.153	0.018	
B_guadalupensis_HT_UF46049	B_guadalupensis_HO_1729	0.002	0.002	gA
B_guadalupensis_JF514630	B_guadalupensis_HO_1729	0.002	0.002	gA
B_sporadicus_FL_1301	B_guadalupensis_HO_1729	0.203	0.021	
B_sp_CO_1414	B_guadalupensis_HO_1729	0.214	0.021	
B_gracilis_AR_1308	B_guadalupensis_HO_1729	0.220	0.021	
B_sporadicus_PA_1316	B_guadalupensis_HO_1729	0.251	0.023	
B_guadalupensis_PR_1707	B_guadalupensis_HO_1729	0.002	0.002	gA
B_guadalupensis_FL_1717	B_guadalupensis_HO_1729	0.002	0.002	gA
B_guadalupensis_DR_1712	B_guadalupensis_HO_1729	0.002	0.002	gA
B_guadalupensis_DO_1722	B_guadalupensis_HO_1729	0.002	0.002	gA
B_guadalupensis_DO_1724	B_guadalupensis_HO_1729	0.002	0.002	gA
B_guadalupensis_DR_1711	B_guadalupensis_HO_1729	0.002	0.002	gA
B_guadalupensis_DR_1714	B_guadalupensis_HO_1729	0.002	0.002	gA
B_guadalupensis_PR_1709	B_guadalupensis_HO_1729	-0.000	0.000	gA
B_guadalupensis_EC_1728	B_guadalupensis_HO_1729	-0.000	0.000	gA
Drymaeus_vexillum_JF514625	B_guadalupensis_PR_1708	0.184	0.019	
Neopetraeus_tesellatus_JF514627	B_guadalupensis_PR_1708	0.158	0.017	
B_hummelincki_JF514629	B_guadalupensis_PR_1708	0.094	0.013	
B_diaphanus_BH_JF514633	B_guadalupensis_PR_1708	0.189	0.020	
B_diaphanus_HT_RMNH_MOL_114274	B_guadalupensis_PR_1708	0.243	0.024	
B_diaphanus_JA_RMNH_MOL_114173	B_guadalupensis_PR_1708	0.244	0.024	
B_diaphanus_SK_RMNH_MOL_114174	B_guadalupensis_PR_1708	0.148	0.018	
B_guadalupensis_HT_UF46049	B_guadalupensis_PR_1708	0.002	0.002	gA
B_guadalupensis_JF514630	B_guadalupensis_PR_1708	0.002	0.002	gA
B_sporadicus_FL_1301	B_guadalupensis_PR_1708	0.205	0.021	
B_sp_CO_1414	B_guadalupensis_PR_1708	0.212	0.021	
B_gracilis_AR_1308	B_guadalupensis_PR_1708	0.218	0.021	
B_sporadicus_PA_1316	B_guadalupensis_PR_1708	0.249	0.023	
B_guadalupensis_PR_1707	B_guadalupensis_PR_1708	0.002	0.002	gA
B_guadalupensis_FL_1717	B_guadalupensis_PR_1708	0.002	0.002	gA
B_guadalupensis_DR_1712	B_guadalupensis_PR_1708	0.002	0.002	gA

B_guadalupensis_DO_1722	B_guadalupensis_PR_1708	0.002	0.002	gA
B_guadalupensis_DO_1724	B_guadalupensis_PR_1708	0.002	0.002	gA
B_guadalupensis_DR_1711	B_guadalupensis_PR_1708	0.002	0.002	gA
B_guadalupensis_DR_1714	B_guadalupensis_PR_1708	0.002	0.002	gA
B_guadalupensis_PR_1709	B_guadalupensis_PR_1708	0.003	0.002	gA
B_guadalupensis_EC_1728	B_guadalupensis_PR_1708	0.003	0.002	gA
B_guadalupensis_HO_1729	B_guadalupensis_PR_1708	0.003	0.002	gA
Drymaeus_vexillum_JF514625	B_guadalupensis_BA_1720	0.195	0.020	
Neopetraeus_tesellatus_JF514627	B_guadalupensis_BA_1720	0.172	0.018	
B_hummelincki_JF514629	B_guadalupensis_BA_1720	0.107	0.014	
B_diaphanus_BH_JF514633	B_guadalupensis_BA_1720	0.203	0.021	
B_diaphanus_HT_RMNH_MOL_114274	B_guadalupensis_BA_1720	0.226	0.023	
B_diaphanus_JA_RMNH_MOL_114173	B_guadalupensis_BA_1720	0.227	0.023	
B_diaphanus_SK_RMNH_MOL_114174	B_guadalupensis_BA_1720	0.155	0.018	
B_guadalupensis_HT_UF46049	B_guadalupensis_BA_1720	0.034	0.007	
B_guadalupensis_JF514630	B_guadalupensis_BA_1720	0.034	0.007	
B_sporadicus_FL_1301	B_guadalupensis_BA_1720	0.203	0.021	
B_sp_CO_1414	B_guadalupensis_BA_1720	0.215	0.021	
B_gracilis_AR_1308	B_guadalupensis_BA_1720	0.228	0.022	
B_sporadicus_PA_1316	B_guadalupensis_BA_1720	0.254	0.023	
B_guadalupensis_PR_1707	B_guadalupensis_BA_1720	0.034	0.007	
B_guadalupensis_FL_1717	B_guadalupensis_BA_1720	0.034	0.007	
B_guadalupensis_DR_1712	B_guadalupensis_BA_1720	0.034	0.007	
B_guadalupensis_DO_1722	B_guadalupensis_BA_1720	0.034	0.007	
B_guadalupensis_DO_1724	B_guadalupensis_BA_1720	0.034	0.007	
B_guadalupensis_DR_1711	B_guadalupensis_BA_1720	0.034	0.007	
B_guadalupensis_DR_1714	B_guadalupensis_BA_1720	0.034	0.007	
B_guadalupensis_PR_1709	B_guadalupensis_BA_1720	0.035	0.008	
B_guadalupensis_EC_1728	B_guadalupensis_BA_1720	0.035	0.008	
B_guadalupensis_HO_1729	B_guadalupensis_BA_1720	0.035	0.008	
B_guadalupensis_PR_1708	B_guadalupensis_BA_1720	0.032	0.007	
Drymaeus_vexillum_JF514625	B_guadalupensis_JA_1727	0.195	0.020	
Neopetraeus_tesellatus_JF514627	B_guadalupensis_JA_1727	0.172	0.018	
B_hummelincki_JF514629	B_guadalupensis_JA_1727	0.107	0.014	
B_diaphanus_BH_JF514633	B_guadalupensis_JA_1727	0.203	0.021	
B_diaphanus_HT_RMNH_MOL_114274	B_guadalupensis_JA_1727	0.226	0.023	
B_diaphanus_JA_RMNH_MOL_114173	B_guadalupensis_JA_1727	0.227	0.023	
B_diaphanus_SK_RMNH_MOL_114174	B_guadalupensis_JA_1727	0.155	0.018	
B_guadalupensis_HT_UF46049	B_guadalupensis_JA_1727	0.034	0.007	
B_guadalupensis_JF514630	B_guadalupensis_JA_1727	0.034	0.007	
B_sporadicus_FL_1301	B_guadalupensis_JA_1727	0.203	0.021	
B_sp_CO_1414	B_guadalupensis_JA_1727	0.215	0.021	
B_gracilis_AR_1308	B_guadalupensis_JA_1727	0.228	0.022	
B_sporadicus_PA_1316	B_guadalupensis_JA_1727	0.254	0.023	
B_guadalupensis_PR_1707	B_guadalupensis_JA_1727	0.034	0.007	

B_guadalupensis_FL_1717	B_guadalupensis_JA_1727	0.034	0.007	
B_guadalupensis_DR_1712	B_guadalupensis_JA_1727	0.034	0.007	
B_guadalupensis_DO_1722	B_guadalupensis_JA_1727	0.034	0.007	
B_guadalupensis_DO_1724	B_guadalupensis_JA_1727	0.034	0.007	
B_guadalupensis_DR_1711	B_guadalupensis_JA_1727	0.034	0.007	
B_guadalupensis_DR_1714	B_guadalupensis_JA_1727	0.034	0.007	
B_guadalupensis_PR_1709	B_guadalupensis_JA_1727	0.035	0.008	
B_guadalupensis_EC_1728	B_guadalupensis_JA_1727	0.035	0.008	
B_guadalupensis_HO_1729	B_guadalupensis_JA_1727	0.035	0.008	
B_guadalupensis_PR_1708	B_guadalupensis_JA_1727	0.032	0.007	
B_guadalupensis_BA_1720	B_guadalupensis_JA_1727	-0.000	0.000	
Drymaeus_vexillum_JF514625	B_guadalupensis_GU_1725	0.202	0.020	
Neopetraeus_tesellatus_JF514627	B_guadalupensis_GU_1725	0.176	0.018	
B_hummelincki_JF514629	B_guadalupensis_GU_1725	0.110	0.014	
B_diaphanus_BH_JF514633	B_guadalupensis_GU_1725	0.217	0.022	
B_diaphanus_HT_RMNH_MOL_114274	B_guadalupensis_GU_1725	0.236	0.023	
B_diaphanus_JA_RMNH_MOL_114173	B_guadalupensis_GU_1725	0.237	0.023	
B_diaphanus_SK_RMNH_MOL_114174	B_guadalupensis_GU_1725	0.164	0.019	
B_guadalupensis_HT_UF46049	B_guadalupensis_GU_1725	0.039	0.008	
B_guadalupensis_JF514630	B_guadalupensis_GU_1725	0.039	0.008	
B_sporadicus_FL_1301	B_guadalupensis_GU_1725	0.215	0.021	
B_sp_CO_1414	B_guadalupensis_GU_1725	0.229	0.022	
B_gracilis_AR_1308	B_guadalupensis_GU_1725	0.230	0.021	
B_sporadicus_PA_1316	B_guadalupensis_GU_1725	0.256	0.023	
B_guadalupensis_PR_1707	B_guadalupensis_GU_1725	0.039	0.008	
B_guadalupensis_FL_1717	B_guadalupensis_GU_1725	0.039	0.008	
B_guadalupensis_DR_1712	B_guadalupensis_GU_1725	0.039	0.008	
B_guadalupensis_DO_1722	B_guadalupensis_GU_1725	0.039	0.008	
B_guadalupensis_DO_1724	B_guadalupensis_GU_1725	0.039	0.008	
B_guadalupensis_DR_1711	B_guadalupensis_GU_1725	0.039	0.008	
B_guadalupensis_DR_1714	B_guadalupensis_GU_1725	0.039	0.008	
B_guadalupensis_PR_1709	B_guadalupensis_GU_1725	0.041	0.008	
B_guadalupensis_EC_1728	B_guadalupensis_GU_1725	0.041	0.008	
B_guadalupensis_HO_1729	B_guadalupensis_GU_1725	0.041	0.008	
B_guadalupensis_PR_1708	B_guadalupensis_GU_1725	0.037	0.008	
B_guadalupensis_BA_1720	B_guadalupensis_GU_1725	0.019	0.006	gB
B_guadalupensis_JA_1727	B_guadalupensis_GU_1725	0.019	0.006	gB
Drymaeus_vexillum_JF514625	B_guadalupensis_DR_1710	0.200	0.020	
Neopetraeus_tesellatus_JF514627	B_guadalupensis_DR_1710	0.176	0.018	
B_hummelincki_JF514629	B_guadalupensis_DR_1710	0.111	0.014	
B_diaphanus_BH_JF514633	B_guadalupensis_DR_1710	0.217	0.022	
B_diaphanus_HT_RMNH_MOL_114274	B_guadalupensis_DR_1710	0.231	0.023	
B_diaphanus_JA_RMNH_MOL_114173	B_guadalupensis_DR_1710	0.232	0.023	
B_diaphanus_SK_RMNH_MOL_114174	B_guadalupensis_DR_1710	0.161	0.019	
B_guadalupensis_HT_UF46049	B_guadalupensis_DR_1710	0.046	0.009	

B_guadalupensis_JF514630	B_guadalupensis_DR_1710	0.046	0.009	
B_sporadicus_FL_1301	B_guadalupensis_DR_1710	0.208	0.021	
B_sp_CO_1414	B_guadalupensis_DR_1710	0.227	0.022	
B_gracilis_AR_1308	B_guadalupensis_DR_1710	0.228	0.022	
B_sporadicus_PA_1316	B_guadalupensis_DR_1710	0.256	0.024	
B_guadalupensis_PR_1707	B_guadalupensis_DR_1710	0.046	0.009	
B_guadalupensis_FL_1717	B_guadalupensis_DR_1710	0.046	0.009	
B_guadalupensis_DR_1712	B_guadalupensis_DR_1710	0.046	0.009	
B_guadalupensis_DO_1722	B_guadalupensis_DR_1710	0.046	0.009	
B_guadalupensis_DO_1724	B_guadalupensis_DR_1710	0.046	0.009	
B_guadalupensis_DR_1711	B_guadalupensis_DR_1710	0.046	0.009	
B_guadalupensis_DR_1714	B_guadalupensis_DR_1710	0.046	0.009	
B_guadalupensis_PR_1709	B_guadalupensis_DR_1710	0.048	0.009	
B_guadalupensis_EC_1728	B_guadalupensis_DR_1710	0.048	0.009	
B_guadalupensis_HO_1729	B_guadalupensis_DR_1710	0.048	0.009	
B_guadalupensis_PR_1708	B_guadalupensis_DR_1710	0.045	0.009	
B_guadalupensis_BA_1720	B_guadalupensis_DR_1710	0.023	0.006	gB
B_guadalupensis_JA_1727	B_guadalupensis_DR_1710	0.023	0.006	gB
B_guadalupensis_GU_1725	B_guadalupensis_DR_1710	0.017	0.005	gB
Drymaeus_vexillum_JF514625	B_corneus_BE_1705	0.237	0.023	
Neopetraeus_tesellatus_JF514627	B_corneus_BE_1705	0.239	0.023	
B_hummelincki_JF514629	B_corneus_BE_1705	0.238	0.024	
B_diaphanus_BH_JF514633	B_corneus_BE_1705	0.149	0.018	
B_diaphanus_HT_RMNH_MOL_114274	B_corneus_BE_1705	0.239	0.023	
B_diaphanus_JA_RMNH_MOL_114173	B_corneus_BE_1705	0.239	0.023	
B_diaphanus_SK_RMNH_MOL_114174	B_corneus_BE_1705	0.208	0.021	
B_guadalupensis_HT_UF46049	B_corneus_BE_1705	0.223	0.022	
B_guadalupensis_JF514630	B_corneus_BE_1705	0.223	0.022	
B_sporadicus_FL_1301	B_corneus_BE_1705	0.212	0.022	
B_sp_CO_1414	B_corneus_BE_1705	0.221	0.022	
B_gracilis_AR_1308	B_corneus_BE_1705	0.257	0.023	
B_sporadicus_PA_1316	B_corneus_BE_1705	0.301	0.025	
B_guadalupensis_PR_1707	B_corneus_BE_1705	0.223	0.022	
B_guadalupensis_FL_1717	B_corneus_BE_1705	0.223	0.022	
B_guadalupensis_DR_1712	B_corneus_BE_1705	0.223	0.022	
B_guadalupensis_DO_1722	B_corneus_BE_1705	0.223	0.022	
B_guadalupensis_DO_1724	B_corneus_BE_1705	0.223	0.022	
B_guadalupensis_DR_1711	B_corneus_BE_1705	0.223	0.022	
B_guadalupensis_DR_1714	B_corneus_BE_1705	0.223	0.022	
B_guadalupensis_PR_1709	B_corneus_BE_1705	0.223	0.022	
B_guadalupensis_EC_1728	B_corneus_BE_1705	0.223	0.022	
B_guadalupensis_HO_1729	B_corneus_BE_1705	0.223	0.022	
B_guadalupensis_PR_1708	B_corneus_BE_1705	0.226	0.022	
B_guadalupensis_BA_1720	B_corneus_BE_1705	0.231	0.023	
B_guadalupensis_JA_1727	B_corneus_BE_1705	0.231	0.023	

B_guadalupensis_GU_1725	B_corneus_BE_1705	0.233	0.023	
B_guadalupensis_DR_1710	B_corneus_BE_1705	0.241	0.024	
Drymaeus_vexillum_JF514625	B_corneus_CR_1706	0.185	0.020	
Neopetraeus_tesellatus_JF514627	B_corneus_CR_1706	0.205	0.021	
B_hummelincki_JF514629	B_corneus_CR_1706	0.203	0.022	
B_diaphanus_BH_JF514633	B_corneus_CR_1706	0.151	0.018	
B_diaphanus_HT_RMNH_MOL_114274	B_corneus_CR_1706	0.206	0.021	
B_diaphanus_JA_RMNH_MOL_114173	B_corneus_CR_1706	0.204	0.021	
B_diaphanus_SK_RMNH_MOL_114174	B_corneus_CR_1706	0.188	0.020	
B_guadalupensis_HT_UF46049	B_corneus_CR_1706	0.191	0.021	
B_guadalupensis_JF514630	B_corneus_CR_1706	0.191	0.021	
B_sporadicus_FL_1301	B_corneus_CR_1706	0.212	0.021	
B_sp_CO_1414	B_corneus_CR_1706	0.211	0.021	
B_gracilis_AR_1308	B_corneus_CR_1706	0.224	0.021	
B_sporadicus_PA_1316	B_corneus_CR_1706	0.266	0.023	
B_guadalupensis_PR_1707	B_corneus_CR_1706	0.191	0.021	
B_guadalupensis_FL_1717	B_corneus_CR_1706	0.191	0.021	
B_guadalupensis_DR_1712	B_corneus_CR_1706	0.191	0.021	
B_guadalupensis_DO_1722	B_corneus_CR_1706	0.191	0.021	
B_guadalupensis_DO_1724	B_corneus_CR_1706	0.191	0.021	
B_guadalupensis_DR_1711	B_corneus_CR_1706	0.191	0.021	
B_guadalupensis_DR_1714	B_corneus_CR_1706	0.191	0.021	
B_guadalupensis_PR_1709	B_corneus_CR_1706	0.191	0.021	
B_guadalupensis_EC_1728	B_corneus_CR_1706	0.191	0.021	
B_guadalupensis_HO_1729	B_corneus_CR_1706	0.191	0.021	
B_guadalupensis_PR_1708	B_corneus_CR_1706	0.189	0.020	
B_guadalupensis_BA_1720	B_corneus_CR_1706	0.198	0.021	
B_guadalupensis_JA_1727	B_corneus_CR_1706	0.198	0.021	
B_guadalupensis_GU_1725	B_corneus_CR_1706	0.202	0.021	
B_guadalupensis_DR_1710	B_corneus_CR_1706	0.202	0.021	
B_corneus_BE_1705	B_corneus_CR_1706	0.160	0.018	
Drymaeus_vexillum_JF514625	B_sporadicus_JF514632	0.237	0.023	
Neopetraeus_tesellatus_JF514627	B_sporadicus_JF514632	0.235	0.022	
B_hummelincki_JF514629	B_sporadicus_JF514632	0.228	0.022	
B_diaphanus_BH_JF514633	B_sporadicus_JF514632	0.241	0.023	
B_diaphanus_HT_RMNH_MOL_114274	B_sporadicus_JF514632	0.238	0.023	
B_diaphanus_JA_RMNH_MOL_114173	B_sporadicus_JF514632	0.238	0.023	
B_diaphanus_SK_RMNH_MOL_114174	B_sporadicus_JF514632	0.240	0.023	
B_guadalupensis_HT_UF46049	B_sporadicus_JF514632	0.228	0.023	
B_guadalupensis_JF514630	B_sporadicus_JF514632	0.228	0.023	
B_sporadicus_FL_1301	B_sporadicus_JF514632	0.221	0.022	
B_sp_CO_1414	B_sporadicus_JF514632	0.243	0.024	
B_gracilis_AR_1308	B_sporadicus_JF514632	0.256	0.024	
B_sporadicus_PA_1316	B_sporadicus_JF514632	0.308	0.027	
B_guadalupensis_PR_1707	B_sporadicus_JF514632	0.228	0.023	

B_guadalupensis_FL_1717	B_sporadicus_JF514632	0.228	0.023	
B_guadalupensis_DR_1712	B_sporadicus_JF514632	0.228	0.023	
B_guadalupensis_DO_1722	B_sporadicus_JF514632	0.228	0.023	
B_guadalupensis_DO_1724	B_sporadicus_JF514632	0.228	0.023	
B_guadalupensis_DR_1711	B_sporadicus_JF514632	0.228	0.023	
B_guadalupensis_DR_1714	B_sporadicus_JF514632	0.228	0.023	
B_guadalupensis_PR_1709	B_sporadicus_JF514632	0.226	0.023	
B_guadalupensis_EC_1728	B_sporadicus_JF514632	0.226	0.023	
B_guadalupensis_HO_1729	B_sporadicus_JF514632	0.226	0.023	
B_guadalupensis_PR_1708	B_sporadicus_JF514632	0.228	0.023	
B_guadalupensis_BA_1720	B_sporadicus_JF514632	0.238	0.024	
B_guadalupensis_JA_1727	B_sporadicus_JF514632	0.238	0.024	
B_guadalupensis_GU_1725	B_sporadicus_JF514632	0.255	0.024	
B_guadalupensis_DR_1710	B_sporadicus_JF514632	0.263	0.025	
B_corneus_BE_1705	B_sporadicus_JF514632	0.253	0.023	
B_corneus_CR_1706	B_sporadicus_JF514632	0.228	0.022	
