# Supplementary material for: Caribbean Bulimulus revisited: physical moves and molecular traces (Mollusca, Gastropoda, Bulimulidae)
Source: PeerJ. 2016 Mar 29;4:e1836. doi: 10.7717/peerj.1836 (PMC4824910; doi:10.7717/peerj.1836)
Supplement: Table S3 [file peerj-04-1836-s005.pdf]

				Closest species	Intra	Inter	Intra/Inter	P ID(strict)	Rosenberg P_{AB}	Rodrigo P(RD)
			ML							
Bulimulus	guadalupensis	PR_1707, …	gA	gB	0.002	0.108	0.02	0.98 (0.92-1.0)	5.2E-05	< 0.05
Bulimulus	guadalupensis	GU_1725, …	gB	gA	0.045	0.108	0.42	0.59 (0.44-0.73)	5.2E-05	1.0
Bulimulus	diaphanus	JA_RMNH.MOL114173	d1	s1	0.003	0.698	0.035	0.59 (0.44-0.74)	0.33	0.14
Bulimulus	diaphanus	SK_RMNH.MOL114174	d2*	gB	0.0	0.047	0.0	0.0	1.0	NA
Bulimulus	diaphanus	BH_ANcP.A22054	d3*	s1	0.0	0.9	0.0	0.0	0.1	NA
Bulimulus	sporadicus	FL_1301	s1*	d1	0.0	0.698	0.0	0.0	0.33	NA
Bulimulus	sporadicus	PA_1316	s2*	d3	0.0	2.148	0.0	0.0	0.07	NA
Bulimulus	sporadicus	TX_JF514633	s3*	s1	0.0	0.992	0.0	0.0	0.17	NA
Bulimulus	guadalupensis	PR_1707, …	gA	gB	0.002	0.111	0.02	0.98 (0.92-1.0)	5.2E-05	< 0.05
Bulimulus	guadalupensis	GU_1725, …	gB	gA	0.046	0.111	0.41	0.59 (0.45-0.73)	5.2E-05	1.0
Bulimulus	diaphanus	JA_RMNH.MOL114173	d1	s1	0.003	0.946	0.0	0.0	0.33	0.12
Bulimulus	diaphanus	SK_RMNH.MOL114174	d2*	hu	0.0	0.716	0.0	0.0	0.01	NA
Bulimulus	diaphanus	BH_ANcP.A22054	d3*	c1	0.0	0.589	0.0	0.0	1.0	NA
Bulimulus	sporadicus	FL_1301	s1*	d1	0.0	0.946	0.0	0.0	0.33	NA
Bulimulus	sporadicus	PA_1316	s2*	s1	0.0	3.497	0.0	0.0	1.0	NA
Bulimulus	sporadicus	TX_JF514633	s3*	s2	0.0	1.282	0.0	0.0	0.17	NA
Bulimulus	hummelincki	JF514632	hu*	gA	0.0	0.386	0.0	0.0	0.01	NA
Bulimulus	corneus	BE_1705	c1*	d3	0.0	0.842	0.0	0.0	0.33	NA
Bulimulus	corneus	CR_1706	c2*	d3	0.0	0.842	0.0	0.0	0.33	NA
Bulimulus	sp.	CO_1414	sp*	s1	0.0	1.4	0.0	0.0	1.0	NA
Bulimulus	gracilis	AR_1308	gr*	d1	0.0	1.978	0.0	0.0	2.46E-03	NA
			BI							
Bulimulus	guadalupensis	PR_1707, …	gA	gB	0.004	0.067	0.06	0.96 (0.90-1.0)	7.3E-05	< 0.05
Bulimulus	guadalupensis	GU_1725, …	gB	gA	0.030	0.067	0.44	0.57 (0.43-0.72)	5.2E-05	< 0.05
Bulimulus	diaphanus	JA_RMNH.MOL114173	d1	s1	0.003	0.350	0.01	0.59 (0.44-0.74)	0.33	< 0.05
Bulimulus	diaphanus	SK_RMNH.MOL114174	d2*	gA	0.0	0.357	0.0	0.0	0.01	NA
Bulimulus	diaphanus	BH_ANcP.A22054	d3*	d2	0.0	0.542	0.0	0.0	3.62E-05	NA
Bulimulus	sporadicus	FL_1301	s1*	d1	0.0	0.350	0.0	0.0	0.33	NA
Bulimulus	sporadicus	PA_1316	s2*	d3	0.0	0.930	0.0	0.0	0.17	NA
Bulimulus	sporadicus	TX_JF514633	s3*	s1	0.0	0.930	0.0	0.0	0.17	NA
Bulimulus	guadalupensis	PR_1707, …	gA	gB	0.004	0.069	0.06	0.96 (0.90-1.0)	5.2E-05	< 0.05
Bulimulus	guadalupensis	GU_1725, …	gB	gA	0.030	0.069	0.43	0.58 (0.43-0.72)	5.2E-05	< 0.05
Bulimulus	diaphanus	JA_RMNH.MOL114173	d1	s1	0.003	0.438	0.01	0.59 (0.44-0.74)	0.33	< 0.05
Bulimulus	diaphanus	SK_RMNH.MOL114174	d2*	hu	0.0	0.381	0.0	0.0	0.01	NA
Bulimulus	diaphanus	BH_ANcP.A22054	d3*	c1	0.0	0.295	0.0	0.0	1.0	NA
Bulimulus	sporadicus	FL_1301	s1*	d1	0.0	0.438	0.0	0.0	0.33	NA
Bulimulus	sporadicus	PA_1316	s2*	c2	0.0	1.279	0.0	0.0	0.17	NA
Bulimulus	sporadicus	TX_JF514633	s3*	s1	0.0	0.598	0.0	0.0	0.03	NA
Bulimulus	hummelincki	JF514632	hu*	gA	0.0	0.208	0.0	0.0	0.01	NA
Bulimulus	corneus	BE_1705	c1*	d3	0.0	0.295	0.0	0.0	1.0	NA
Bulimulus	corneus	CR_1706	c2*	d3	0.0	0.295	0.0	0.0	1.0	NA
Bulimulus	sp.	CO_1414	sp*	s1	0.0	0.566	0.0	0.0	0.03	NA
Bulimulus	gracilis	AR_1308	gr*	d1	0.0	0.931	0.0	0.0	2.46E-03	NA
