# Supplementary material for: Caribbean Bulimulus revisited: physical moves and molecular traces (Mollusca, Gastropoda, Bulimulidae)
Source: PeerJ. 2016 Mar 29;4:e1836. doi: 10.7717/peerj.1836 (PMC4824910; doi:10.7717/peerj.1836)
Supplement: Table S4 [file peerj-04-1836-s006.pdf]

			MeanDistances		gp1	gp2	gp3	gp4	gp5	gp6	gp7	gp8	gp9	gp10	gp11	gp12
Bulimulus	guadalupensis	PR_1707, …	gA	gp1												
Bulimulus	guadalupensis	GU_1725, …	gB	gp1	0.016 (0.003)											
Bulimulus	diaphanus	JA_RMNH.MO L114173	d1	gp2												
Bulimulus	diaphanus	SK_RMNH.MO L114174	d2*	gp2	0.206 (0.018)	0.194 (0.017)										
Bulimulus	diaphanus	BH_ANcP.A22 054	d3*	gp2												
Bulimulus	sporadicus	FL_1301	s1*	gp3												
Bulimulus	sporadicus	PA_1316	s2*	gp3	0.229 (0.018)	0.240 (0.017)	0.268 (0.021)									
Bulimulus	sporadicus	TX_JF514633	s3*	gp3												
Bulimulus	hummelincki	JF514632	hu*	gp5	0.098 (0.013)	0.217 (0.019)	0.236 (0.018)	0.225 (0.018)	n/c							
Bulimulus	corneus	BE_1705	c1*	gp6												
Bulimulus	corneus	CR_1706	c2*	gp6	0.210 (0.019)	0.198 (0.016)	0.245 (0.018)	0.228 (0.017)	0.221 (0.020)	0.160 (0.019)						
Bulimulus	sp.	CO_1414	sp*	gp4												
Bulimulus	gracilis	AR_1308	gr*	gp4	0.218 (0.017)	0.243 (0.018)	0.256 (0.017)	0.272 (0.026)								
			MeanDistances													
Bulimulus	guadalupensis	PR_1707, …	gA	gp1	0.001 (0.001)											
Bulimulus	guadalupensis	GU_1725, …	gB	gp2	0.039 (0.007)	0.017 (0.004)										
Bulimulus	diaphanus	JA_RMNH.MO L114173	d1	gp3	0.241 (0.022)	0.230 (0.021)	n/c									
Bulimulus	diaphanus	SK_RMNH.MO L114174	d2*	gp4	0.151 (0.017)	0.159 (0.018)	0.258 (0.024)	n/c								
Bulimulus	diaphanus	BH_ANcP.A22 054	d3*	gp5	0.187 (0.020)	0.210 (0.022)	0.255 (0.020)	0.195 (0.023)	n/c							
Bulimulus	sporadicus	FL_1301	s1*	gp6	0.203 (0.021)	0.207 (0.021)	0.182 (0.020)	0.223 (0.023)	0.179 (0.020)	n/c						
Bulimulus	sporadicus	PA_1316	s2*	gp7	0.251 (0.023)	0.255 (0.023)	0.283 (0.025)	0.310 (0.026)	0.280 (0.026)	0.274 (0.025)	n/c					
Bulimulus	sporadicus	TX_JF514633	s3*	gp8	0.227 (0.023)	0.248 (0.024)	0.238 (0.024)	0.240 (0.022)	0.241 (0.023)	0.221 (0.023)	0.308 (0.027)	n/c				
Bulimulus	hummelincki	JF514632	hu*	gp9	0.094 (0.014)	0.109 (0.014)	0.148 (0.023)	0.148 (0.014)	0.212 (0.022)	0.212 (0.022)	0.268 (0.025)	0.228 (0.022)	n/c			
Bulimulus	corneus	BE_1705	c1*	gp10												
Bulimulus	corneus	CR_1706	c2*	gp10	0.207 (0.020)	0.217 (0.020)	0.222 (0.021)	0.198 (0.018)	0.150 (0.016)	0.212 (0.020)	0.284 (0.023)	0.240 (0.021)	0.221 (0.020)	0.160 (0.019)		
Bulimulus	sp.	CO_1414	sp*	gp11	0.213 (0.021)	0.221 (0.021)	0.229 (0.025)	0.231 (0.022)	0.231 (0.023)	0.195 (0.021)	0.276 (0.025)	0.243 (0.024)	0.210 (0.021)	0.216 (0.020)	n/c	
Bulimulus	gracilis	AR_1308	gr*	gp12	0.220 (0.021)	0.228 (0.020)	0.275 (0.025)	0.215 (0.020)	0.262 (0.025)	0.254 (0.023)	0.254 (0.026)	0.256 (0.025)	0.240 (0.020)	0.240 (0.021)	0.272 (0.026)	n/c
