# Supplementary material for: Caribbean Bulimulus revisited: physical moves and molecular traces (Mollusca, Gastropoda, Bulimulidae)
Source: PeerJ. 2016 Mar 29;4:e1836. doi: 10.7717/peerj.1836 (PMC4824910; doi:10.7717/peerj.1836)
Supplement: Table S5 [file peerj-04-1836-s007.pdf]

				ML				BI		
				grp1-3	grp1-7	grp1-5	grp1-6	grp1-3	grp1-5	grp1-6
Bulimulus	guadalupensis	PR_1707	gA	grp 1	grp 1	grp 1	grp 1	grp 1	grp 1	grp 1
Bulimulus	guadalupensis	PR_1708	gA	grp 1	grp 1	grp 1	grp 1	grp 1	grp 1	grp 1
Bulimulus	guadalupensis	PR_1709	gA	grp 1	grp 1	grp 1	grp 1	grp 1	grp 1	grp 1
Bulimulus	guadalupensis	DR_JF514630	gA	grp 1	grp 1	grp 1	grp 1	grp 1	grp 1	grp 1
Bulimulus	guadalupensis	DR_1710	gB	grp 1	grp 1	grp 1	grp 2	grp 1	grp 1	grp 2
Bulimulus	guadalupensis	DR_1711	gA	grp 1	grp 1	grp 1	grp 1	grp 1	grp 1	grp 1
Bulimulus	guadalupensis	DR_1712	gA	grp 1	grp 1	grp 1	grp 1	grp 1	grp 1	grp 1
Bulimulus	guadalupensis	DR_1714	gA	grp 1	grp 1	grp 1	grp 1	grp 1	grp 1	grp 1
Bulimulus	guadalupensis	FL_1717	gA	grp 1	grp 1	grp 1	grp 1	grp 1	grp 1	grp 1
Bulimulus	guadalupensis	BA_1720	gB	grp 1	grp 1	grp 1	grp 2	grp 1	grp 1	grp 2
Bulimulus	guadalupensis	DO_1722	gA	grp 1	grp 1	grp 1	grp 1	grp 1	grp 1	grp 1
Bulimulus	guadalupensis	DO_1724	gA	grp 1	grp 1	grp 1	grp 1	grp 1	grp 1	grp 1
Bulimulus	guadalupensis	GU_1725	gB	grp 1	grp 1	grp 1	grp 2	grp 1	grp 1	grp 2
Bulimulus	guadalupensis	JA_1727	gB	grp 1	grp 1	grp 1	grp 2	grp 1	grp 1	grp 2
Bulimulus	guadalupensis	EC_1728	gA	grp 1	grp 1	grp 1	grp 1	grp 1	grp 1	grp 1
Bulimulus	guadalupensis	HO_1729	gA	grp 1	grp 1	grp 1	grp 1	grp 1	grp 1	grp 1
Bulimulus	guadalupensis	HT_UF46049	gA	grp 1	grp 1	grp 1	grp 1	grp 1	grp 1	grp 1
Bulimulus	diaphanus	JA_RMNH.MO<br>L114173	d1	grp 3	grp 3	grp 3	grp 4	grp 3	grp 3	grp 4
Bulimulus	diaphanus	SK_RMNH.MO<br>L114174	d2	grp 3	grp 2	grp 2	grp 3	grp 3	grp 2	grp 3
Bulimulus	diaphanus	HT_RMNH.MO<br>L114274	d1	grp 3	grp 3	grp 3	grp 4	grp 3	grp 3	grp 4
Bulimulus	diaphanus	BH_ANSP.A22<br>054	d3	grp 3	grp 4	grp 3	grp 3	grp 3	grp 3	grp 3
Bulimulus	sporadicus	FL_1301	s1	grp 2	grp 5	grp 4	grp 5	grp 2	grp 4	grp 5
Bulimulus	sporadicus	PA_1316	s2	grp 2	grp 6	grp 4	grp 6	grp 2	grp 4	grp 6
Bulimulus	sporadicus	TX_JF514633	s3	grp 2	grp 7	grp 5	grp 5	grp 2	grp 5	grp 5
			grp 1	GSI=1;<br>P=0.0001	GSI=1;<br>P=0.0001	GSI=1;<br>P=0.0001	GSI=1;<br>P=0.0001	GSI=1;<br>P=0.0001	GSI=1;<br>P=0.0001	GSI=1;<br>P=0.0001
			grp 2	GSI=0.2208;<br>P=0.1426			GSI=1;<br>P=0.0008	GSI=0.3469;<br>P=0.0083		GSI=1;<br>P=0.0003
			grp 3	GSI=0.7143;<br>P=0.0015	GSI=1;<br>P=0.0168	GSI=0.4545;<br>P=0.0256	GSI=0.2174;<br>P=0.2217	GSI=0.6364;<br>P=00.37	GSI=0.2727;<br>P=0.0245	GSI=0.3043;<br>P=0.0656
			grp 4			GSI=0.4783;<br>P=0.0667	GSI=1;<br>P=0.0147		GSI=0.4783;<br>P=0.0324	GSI=1;<br>P=0.0095
			grp 5				GSI=0.4783;<br>P=0.0683			GSI=0.3043;<br>P=0.0684
